# tappAS: a comprehensive computational framework for the analysis of the functional impact of differential splicing

**DOI:** 10.1186/s13059-020-02028-w

**Published:** 2020-05-18

**Authors:** Lorena de la Fuente, Ángeles Arzalluz-Luque, Manuel Tardáguila, Héctor del Risco, Cristina Martí, Sonia Tarazona, Pedro Salguero, Raymond Scott, Alberto Lerma, Ana Alastrue-Agudo, Pablo Bonilla, Jeremy R. B. Newman, Shunichi Kosugi, Lauren M. McIntyre, Victoria Moreno-Manzano, Ana Conesa

**Affiliations:** 1 Genomics of Gene Expression Laboratory, Prince Felipe Research Center, Valencia, Spain; 2 Present Address: Bioinformatics Unit, IIS Fundación Jiménez Díaz, Madrid, Spain; 3 Department of Statistics and Operational Research, Polytechnical University of Valencia, Valencia, Spain; 4 Department of Microbiology and Cell Science, Institute for Food and Agricultural Sciences, University of Florida, Gainesville, FL USA; 5 Present Address: Human Genetics Department, Wellcome Trust Sanger Institute, Hinxton, Cambridge, UK; 6 Genetics Institute, University of Florida, Gainesville, FL USA; 7 Department of Pathology, University of Florida, Gainesville, FL USA; 8 Laboratory for Statistical and Translational Genetics, Center for Integrative Medical Sciences, RIKEN, Wako, Japan; 9 Department of Molecular Genetics and Microbiology, University of Florida, Gainesville, FL USA; 10 Neural Regeneration Laboratory, Prince Felipe Research Center, Valencia, Spain

## Abstract

Recent advances in long-read sequencing solve inaccuracies in alternative transcript identification of full-length transcripts in short-read RNA-Seq data, which encourages the development of methods for isoform-centered functional analysis. Here, we present tappAS, the first framework to enable a comprehensive Functional Iso-Transcriptomics (FIT) analysis, which is effective at revealing the functional impact of context-specific post-transcriptional regulation. tappAS uses isoform-resolved annotation of coding and non-coding functional domains, motifs, and sites, in combination with novel analysis methods to interrogate different aspects of the functional readout of transcript variants and isoform regulation. tappAS software and documentation are available at https://app.tappas.org.

## Introduction

One of the most exciting aspects of transcriptome biology is the contextual adaptability of eukaryotic transcriptomes and proteomes by alternative splicing (AS), alternative polyadenylation (APA), and alternative transcription start sites (ATSS), jointly referred to here as alternative transcript expression mechanisms (AltTEM). These three mechanisms determine which transcripts and isoforms are produced for a given gene in a given context. Alternative transcripts differ in structure and may also differ in function, cell specificity, and spatio-temporal deployment.

The structure and functionality of specific isoforms from single genes have been demonstrated [1, 2]⁠, and while some discrepancy exists on the magnitude of the functional role of isoform diversity [3, 4], AltTEM have been proven to be implicated in a large diversity of cellular processes such as differentiation [5–7]⁠, tissue identity [8, 9]⁠, development [10, 11]⁠, stress response [12], and disease [13–18]. Moreover, several studies have shown the enrichment of spliced exons in disordered regions mediating protein interactions [19–21]⁠, and AS has been found to regulate domains leading to the rewiring of PPI networks in cancer [22]⁠. Similarly, APA has been postulated as a mechanism to escape microRNA regulation by shortening 3′ UTR regions [23, 24]⁠, alternative TSS are believed to regulate the inclusion of upstream open reading frames (uORFs) that control translational rates [25–27]⁠, and non-sense-mediated decay (NMD) has been proposed to regulate gene expression in cancer and neural systems [28, 29]⁠. In parallel, molecular studies have revealed the mechanisms behind the dynamic changes in splicing patterns, identifying a large number of RNA-binding proteins (RBPs) as regulators of AS [11, 30–34].

The study of AltTEM has also been addressed by data-intensive computational methods that established the biological context dependence of alternative isoform expression on a genome-wide scale [35, 36]⁠. Computational AltTEM analyses have devoted much attention to the study of processing *events*, such as changes in the inclusion/exclusion levels of different exons and introns or in the usage of alternative transcription start (TSS) and termination (TTS) sites, as well as NMD mechanisms [37–40]. Consequently, much effort in the field of bioinformatics has been dedicated to analyze and interpret the structural, functional, and regulatory aspects of these individual alternative events [41–43]⁠. However, there is a substantial gap regarding computational approaches dedicated to elucidating the functional implications of AltTEM in a context-specific manner. Methods for functional transcriptome profiling such as enrichment and network analysis [44–47], which have been instrumental for the characterization of transcriptome biology, are routinely operated at the gene level and do not include testing for potential functional differences among isoforms. The field therefore lacks a comprehensive approach to identify and characterize the functional role of AltTEM aside from some examples restricted to certain types of events or particular biological systems [48–53]. Recently, Exon Ontology [54] was proposed as a resource to study functional enrichment of exon sets based on their annotation with protein functional domains. Using this tool, authors established that different molecular functionalities are directly associated to changes in exon inclusion levels between epithelial and mesenchymal cells. However, this analysis neither reveals how transcripts combine exons to generate distinct functional properties nor addresses the analysis of regulatory signals within alternative UTRs. Other software tools aimed at integrating isoform-level analyses with a functional perspective, such as IsoformSwitchAnalyzeR [55], are limited to transcript expression switches, leave other aspects of isoform regulation unaddressed, and fully rely on external tools to provide functional insight.

The inability of RNA-Seq to accurately capture the expression of alternative isoforms [56] is one of the main reasons behind the lack of functional perspective of current splicing bioinformatic tools and their event-oriented approach⁠. Recently, the emergence of third-generation sequencing technologies has proven to be a solution for full-length transcript sequencing [57–61], enabling precise discrimination of different expressed alternative isoforms. Quantification of expression levels is then achieved either by mapping short-read sequencing to this full-length transcriptome [60]⁠ or by the utilization of new high-throughput instruments, which have the potential to allow long read-based study of transcriptome dynamics. As expression studies using these new platforms increase, there will be a growing need for tools that make it easier and quicker to interpret isoform usage changes in the context of their potential functional impact.

Here, we present a novel computational framework for the functional study of AltTEM, introducing the Functional Iso-Transcriptomics (FIT) analysis approach. This framework integrates data from multiple functional databases and tools to create isoform-level annotations of domains, motifs, and sites—both coding and non-coding-, which are then combined with novel metrics and analysis methods to interrogate different aspects of the functional properties associated to the isoform expression dynamics. These methods are implemented in the tappAS (https://app.tappas.org), a user-friendly Java application that brings FIT directly into the hands of researchers working on both model and non-model species. In this study, we aim to illustrate the power of this framework by using tappAS to compare the isoform-quantified transcriptomes of two mouse neural cell types, neural precursor cells (NPCs) and oligodendrocyte precursor cells (OPCs), which were defined with PacBio long reads and quantified using Illumina sequencing in a previous study [62]. We demonstrate how different tappAS analyses recapitulate a great deal of the extant knowledge of AltTEM function in neural tissues and provide novel functional insights amenable to experimental validation. We anticipate that tappAS’ user-friendliness will promote the adoption of the FIT approach by researchers with different levels of computational skills and working across a variety of organisms.

## Results

### tappAS is a comprehensive tool to investigate potential functional consequences of AltTEM

**Fig. 1:**
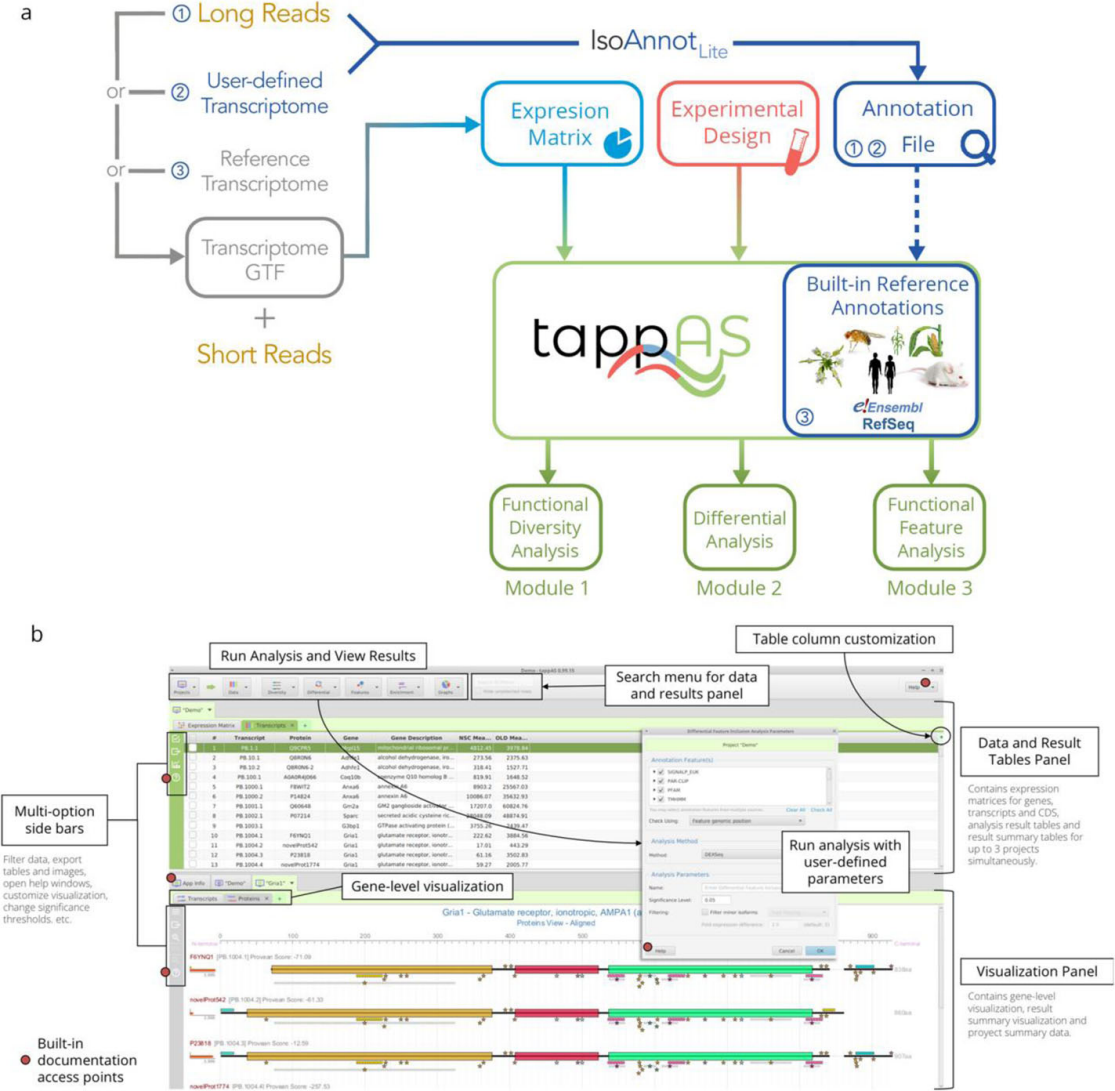
Overview tappAS usage. **a** tappAS project creation workflow. tappAS requires three input files: a transcript-level expression matrix, an experimental design file, and a transcript-level functional annotation file. The expression matrix is typically obtained by mapping RNA-Seq short reads to either a reference transcriptome, a user-defined transcriptome, or an experiment-specific, full-length transcriptome obtained by long-read sequencing. In the last two last cases, the functional annotation file needs to be created using the IsoAnnot_Lite_ script and provided as input upon project creation. When the reference transcriptome is used, users may select tappAS built-in annotation files. **b** The main features of tappAS graphical user interface (GUI)

tappAS is a graphical user interface (GUI) application written in Java and conceived as a user-friendly and flexible framework to enable FIT analysis. The application has a low number of dependencies (Java ≥ v8.40 and R ≥ 3.2.2 are sufficient to run tappAS on any computer, regardless of its operating system) and ensures complete automation of the installation process. Once the main application is downloaded and executed, tappAS manages the installation of all required R packages, as well as the download of the necessary annotation files. A key feature in tappAS is its built-in functional annotation data, which includes positionally defined functional labels (domains, sites, and motifs) from species-available databases and sequence-based prediction algorithms. These annotations provide an extensive functional characterization of both coding and non-coding elements with transcript-level resolution (Table S1). Currently, tappAS includes transcript- and protein isoform-level annotations for humans, mice, Arabidopsis, flies, and maize, ranging from up to 2.5 million of positional functional features from 17 databases in mice (average, 24 features/transcript) to 1 million and 14, respectively, in maize (average, 7 features/transcript) (Table S2). A tappAS project requires three inputs: an experimental design file, a transcript expression matrix, and a transcript-level functional annotation file (Fig. 1a). The transcript expression matrix can be derived from short-read mapping and quantification using either a reference transcriptome, a user-defined transcript set, or a long read-defined, experiment-specific transcriptome, although the latter is recommended to maximize transcriptome definition accuracy. In the first case, and when working with application-supported species (Fig. 1a), tappAS’ built-in annotation data is used and no annotation files are required. In the case of long-read or user-defined transcriptomes, the possible presence of novel isoforms imposes the de novo annotation of the transcript dataset. For this purpose, tappAS toolkit includes the IsoAnnot_Lite_ algorithm (https://isoannot.tappas.org), which positionally maps tappAS functional annotation labels onto newly defined transcriptomes (Fig. 1a). Transcript expression can be inputted as either normalized values or raw counts. In the case of raw counts, tappAS provides the option to apply TMM normalization [63] upon project creation. Finally, tappAS offers an intuitive and easy-to-use graphical user interface with built-in software documentation (Fig. 1b) and multiple options for data export, for filtering, and for combining different analysis modules. Video tutorials illustrating these options are available at https://app.tappas.org.

**Fig. 2:**
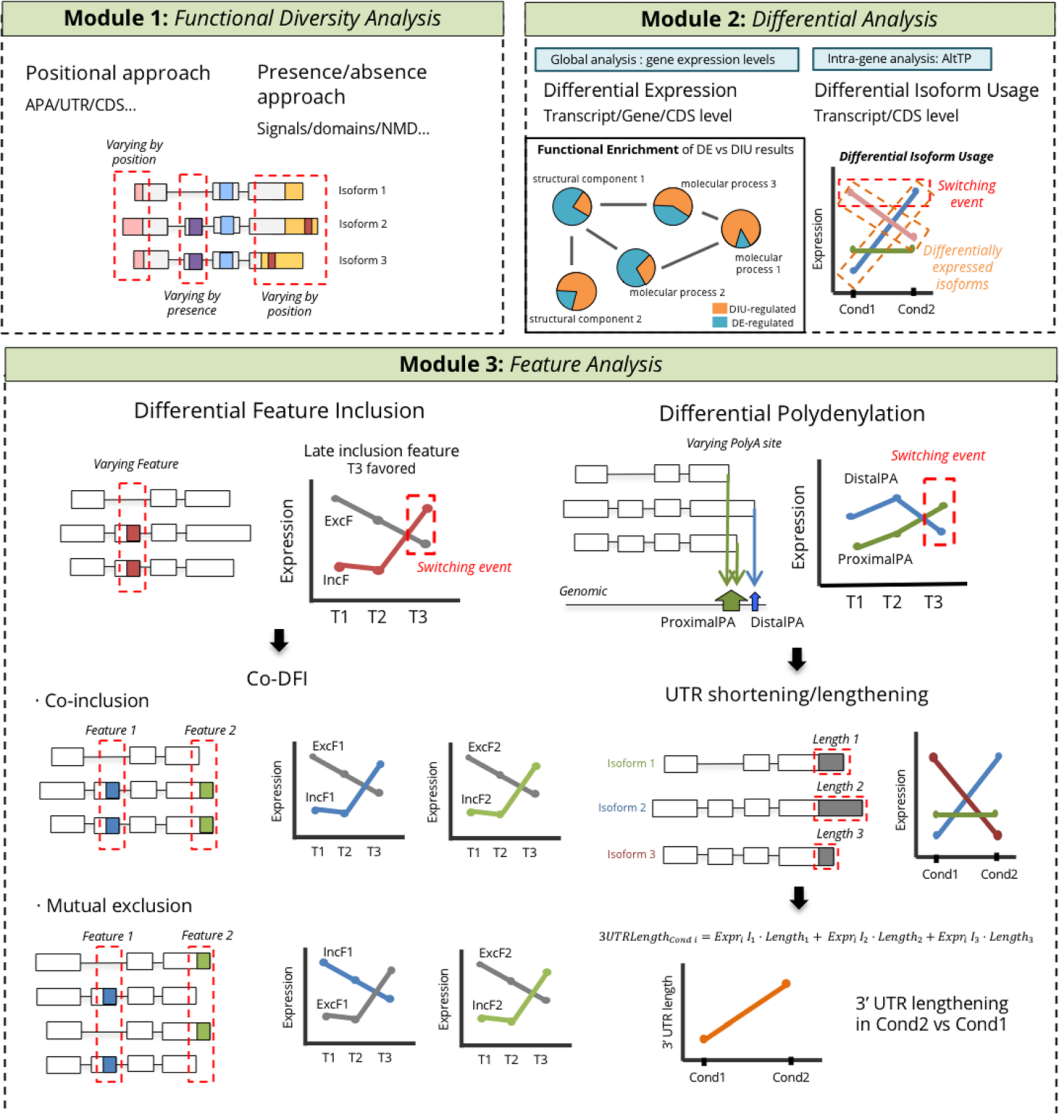
Overview of tappAS modules for Functional Iso-Transcriptomics analysis**.** Module I contains a novel qualitative approach to evaluate the functional diversity of alternative isoforms. Module II implements differential expression and differential isoform usage analyses to compare AltTEM and gene expression. Module 3 includes newly developed approaches to measure the functional impact of AltTEM as changes in the inclusion of functional features, polyA site usage, and UTR length

tappAS integrates transcript-level expression data and this extensive functional annotation with a wide array of traditional and novel analysis approaches (Table 1) to create a comprehensive framework for the study of the functional impact of AltTEM. tappAS analyses can be divided into three modules, each one targeting a different aspect in the study of the AltTEM biology (Fig. 2). Module I implements functional diversity analysis (FDA), which evaluates the functional regulatory potential of AltTEM by interrogating whether features vary across isoforms from the same gene (*varying* status) (Fig. 2, the “Methods” section). This includes analysis by gene, i.e., assessing the varying status of individual genes for each functional feature category, and by feature ID, i.e., assessing the number of genes for which a particular feature is differentially present across isoforms. Varying status is computed both by genomic position (positional approach) and by the presence/absence (presence approach)—depending on the type of feature under evaluation (Table 1, the “Methods” section)—and global varying rates are computed for each functional category. Module II can be used to explore the relative contribution of transcriptional and post-transcriptional regulation in the system under study by comparing differential isoform usage (DIU, transcript level) or differential coding sequence usage (DCU, protein level) with differential gene expression (DGE) results. These are further illustrated by several novel metrics of the magnitude of isoform-level changes (Table 1). Finally, module III includes methods to assess the context-dependent usage of annotated functional elements: differential feature inclusion (DFI) of coding and non-coding elements, differential polyadenylation (DPA), and 3′ and 5′ UTR lengthening analysis (3/5UL). Furthermore, a subsequent co-differential feature inclusion (co-DFI) analysis can detect sets of features that are coordinately included or mutually excluded. Of note, all analysis outputs above can be coupled to functional enrichment [64] and gene set enrichment [65] analyses, which can in turn be based on any of the categories included in tappAS’ annotation for an extended functional readout. Finally, we have made a strong asset of tappAS to provide a readily interpretable visualization interface, featuring comprehensive graphical maps of all annotated features at the genomic, transcript, and protein isoform levels. Further details are available in the “Methods” section.

**Table 1:**
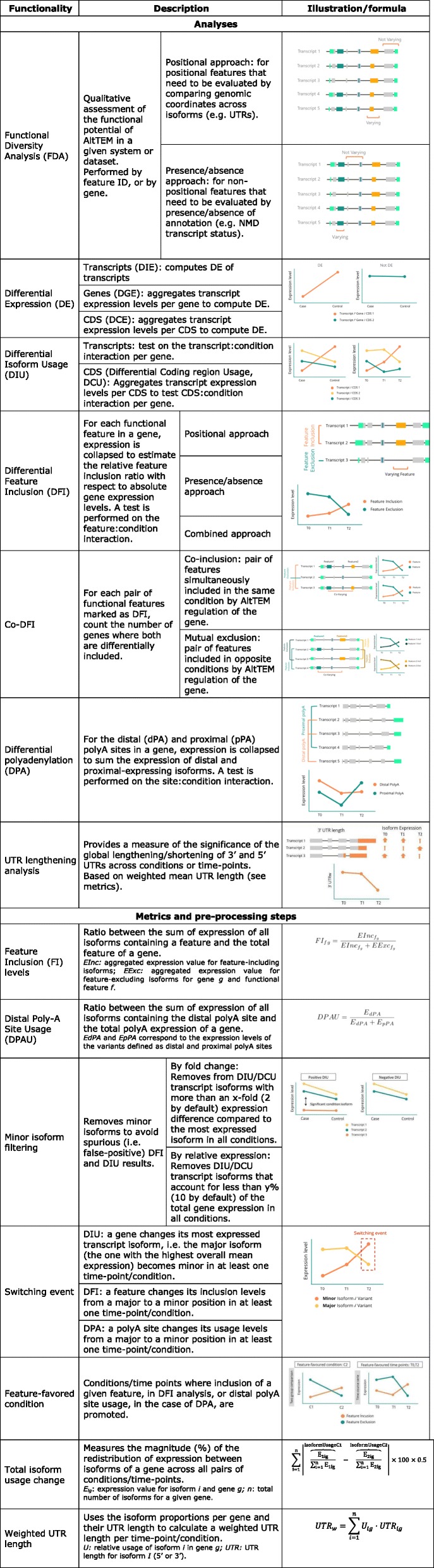
The main analyses and metrics of tappAS

To illustrate tappAS’ analyses, we use the transcriptomics dataset described in Tardaguila et al. [62], consisting of two different mouse cell types, neural precursor cells (NPC) from the spinal cord and oligodendrocyte progenitor cells (OPCs) obtained by in vitro differentiation of NPCs. Two biological replicates for each cell type were sequenced both by PacBio and Illumina. PacBio reads were used to generate an experiment-specific transcriptome that, after SQANTI QC filtering [62], contained 11,970 transcripts from 7167 genes. Transcripts were quantified by Illumina reads using RSEM [66], and raw counts were loaded into tappAS and normalized by TMM [63] and sequencing depth [67]. Additionally, transcripts were annotated with functional sites, motifs, and domains (see the “Methods” section) to a total of 386,114 annotations (Table S3).

### Functional diversity analysis

The potential of AltTEM to regulate gene function is fully illustrated by the variation in functional elements annotated across transcript and protein isoforms. One fundamental question about AltTEM is to what extent post-transcriptional regulation impacts the potential functional complexity of transcriptomes. Functional diversity analysis (FDA) catalogs the functional diversity in the transcriptome annotation and qualitatively assesses changes in these labels across isoforms from the same gene.

**Fig. 3:**
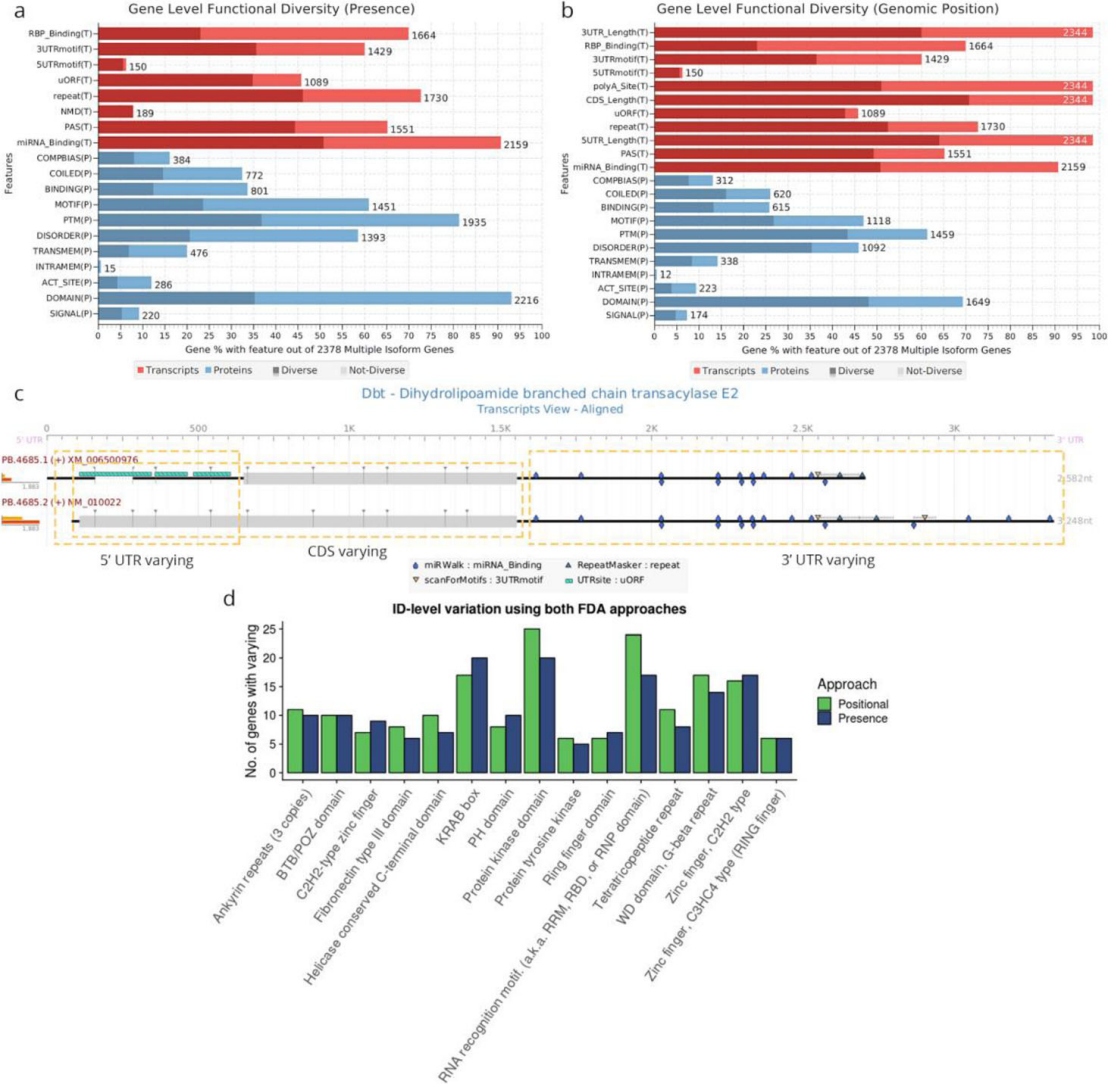
Functional diversity analysis (FDA) results. Annotation type summary of FDA results for the murine neural dataset with the presence/absence (**a**) and positional (**b**) approaches. The percentage of multi-isoform genes annotated with the feature type is shown by total bar length, where the light and dark areas indicate respectively the percentage of genes where none or at least one isoform is varying for that feature type. The numbers above the bars indicate the total no. of feature-containing genes for that category. Both transcript (red) and protein/coding (blue) feature categories are shown. **c** tappAS graphical representation of the transcript-level annotation for the *Dbt* gene, where 5′ UTR, CDS, and 3′/alternative polyadenylation variation were detected. Dotted lines indicate the varying regions. **d** Comparison of position vs the presence/absence approach FDA results for the ID-level analysis of variation in PFAM domains. Top 15 domain families ranked by the total number of varying genes are shown

Applied to our murine neural transcriptomes, FDA using the presence/absence approach identified over 50% of multi-isoform genes with varying status for most annotation types considered, despite the fact that the total number of annotated features differed across categories (Fig. 3a). Among them, non-sense-mediated decay (NMD) was the transcript-level feature with the highest rate of varying (95%), indicating that nearly all genes that generate NMD candidate isoforms also express functional counterparts. UTR motif-annotated genes showed the presence/absence rate of varying 55% and 90% for 3′ and 5′ UTR motifs, respectively (Fig. 3a). Regarding protein-level features, the presence/absence FDA indicated that signal peptides had the highest rate of varying (~ 50%), followed by compositional bias regions and post-translational modifications (PTMs) (Fig. 3a).

In addition to evaluating broad annotation categories, FDA can also be run for all feature IDs within each category in order to reveal which particular functional features tend to have more variability (see Table 1 and the “Methods” section). In our mouse transcriptome, ID-level FDA identified GU-rich elements (GREs) as the most significantly varying 3′ UTR motif (Table S4). GREs have been associated to the stabilization of mRNAs [68] and have also been reported to be targets of RNA-binding proteins (RBPs) such as CELFs [69]⁠. Among the set of 160 genes where GREs varied across isoforms, FDA identified splicing regulators such as *Rbm4* (Figure S1A), involved in neurogenesis of the mouse embryonic brain [70]⁠, and *Tcf12* (Figure S1B), known to play an important role in the control of proliferating neural stem cells and progenitor cells during neurogenesis [71]⁠.

ID-level FDA also identified a large number (508) of varying miRNA binding sites at the 3′ UTR. The top five miRNAs (Table S5) included miR-335-3p, known to associate with oligodendrocyte differentiation [72], and mir-590-3p, which responds to retinoic acid and is strongly associated to proliferation and differentiation processes [73, 74]⁠. Since NPCs and OPCs constitute differentiating cells, these results suggest a potential AltTEM-based isoform-specific regulatory mechanism via gain and loss of miRNA binding sites in oligodendrocyte differentiation.

FDA can be refined by applying the positional varying approach (see Table 1 and the “Methods” section), which can detect subtle changes in feature encoding (as opposed to complete feature disruption) as well as analyze structural categories that are solely positionally defined, such as CDS and UTR length variation (Fig. 3b). Using the positional approach, FDA identified ~ 70% out of 2341multi-isoform genes showing variation in predicted proteins (CDS) length, while variability in 3′ and 5′ UTR lengths occurred in ~ 60% of the genes (Fig. 3b). Interestingly, the vast majority (78%) of UTR-varying genes also had CDS variation, linking protein diversity to RNA regulatory diversity. To illustrate, Fig. 3c shows an example of a gene detected by FDA and visualized in tappAS as alternative polyadenylation (APA), 5′ UTR, 3′ UTR, and CDS-varying. Besides transcript-level features, the FDA positional approach can be particularly useful to discriminate protein motifs and domains that are not completely skipped, but only miss partial sequences. For protein-coding features, results showed that intrinsically disordered regions (IDRs) (which have been previously reported to be enriched in transcript regions affected by AltTEM [19, 75, 76]) and PFAM domains presented the highest variation rates in multi-isoform genes annotated for these feature categories (~ 78% and ~ 70%, respectively; Fig. 3b). Figure 3d shows the top 15 PFAM domains ranked by variation rate in the murine neural data. Positional vs presence results in zinc fingers and KRAB-box domains indicate that these domains tend to be totally contained in alternatively processed regions, given that the varying rate reported using both approaches is similar. Hence, domain skipping in these cases will likely result in elimination from the protein. In contrast, positional FDA results in kinase- and RNA-binding domains indicate that AltTEM mechanisms partially modify these domains, possibly causing modulation of function.

In summary, tappAS’ implementation of FDA is able to recapitulate the AltTEM-mediated functional diversity of transcriptomes and, in our murine neural system, shows that ~ 90% of multi-isoform genes have protein or transcript-level features varying across isoforms for the considered set of functional annotation categories.

### Differential transcript-level analysis

Transcription and AltTEM regulate gene activity by controlling the total gene expression and the relative levels of their alternative isoforms, respectively. These two processes can be computationally assessed by differential gene expression (DE) and differential isoform usage (DIU) analyses. Statistical significance in DE is frequently complemented by a measure of potential biological relevance (i.e., fold change); however, the corresponding estimate of biological impact has not been developed for DIU. Module II in tappAS incorporates these two analyses, complemented with methods to select DIU changes based on switching events and total isoform usage change. Furthermore, the framework allows both regulatory layers to be compared from a functional perspective.

**Fig. 4:**
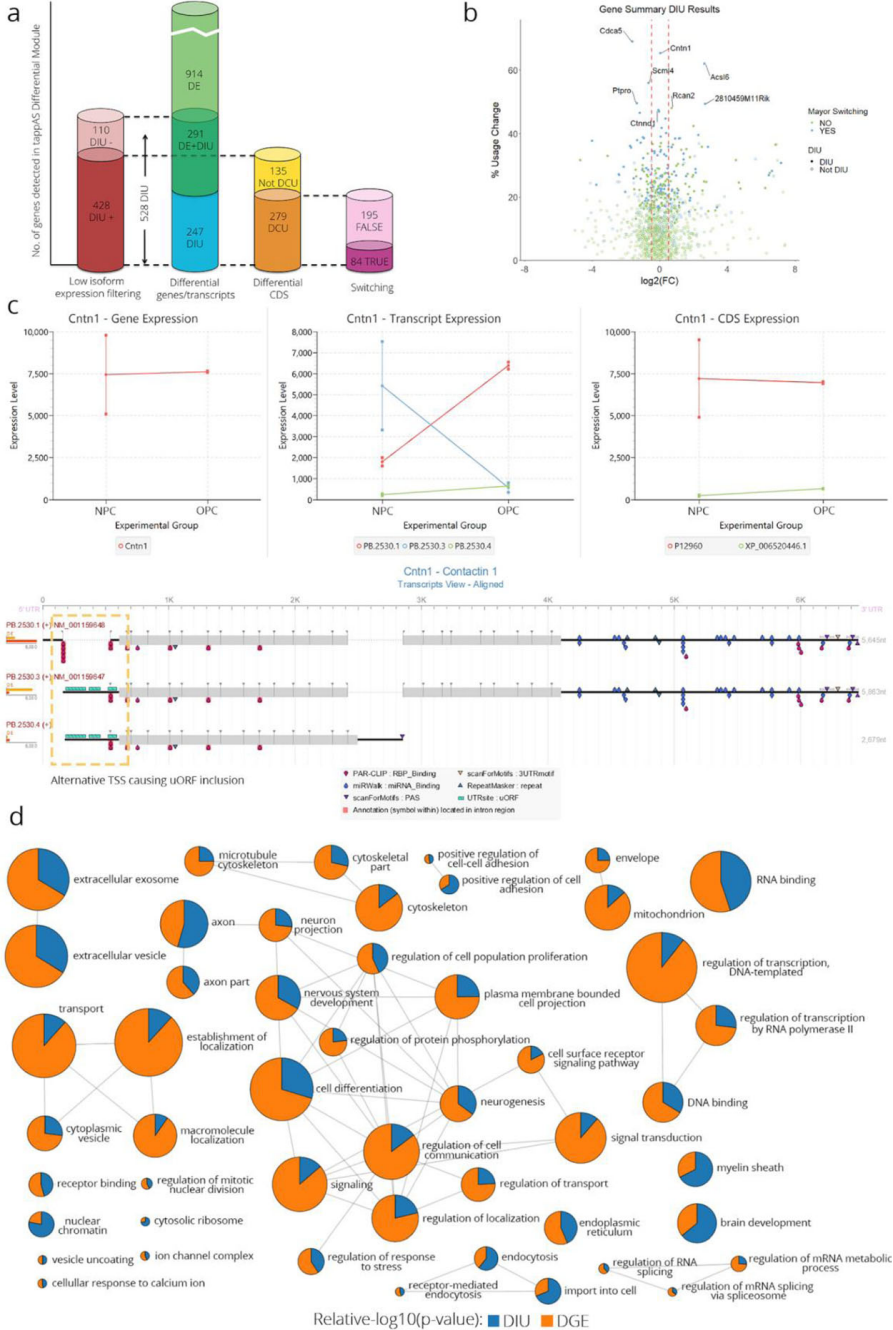
Combined analysis of differential gene expression and AltTEM in tappAS**. a** From left to right: intersections of differential module results: DIU with and without minor isoform filtering (eliminates isoforms with gene proportion of expression < 0.1) of DE vs DIU and DIU vs DCU and major isoform switching vs no switching. Numbers and vertical bar heights indicate the number of genes in each group. **b** Total usage change (i.e., expression redistribution between isoforms) vs log-transformed values of gene expression fold change between cell types. Genes with a major isoform switch are represented in orange. Labels are assigned to genes with the highest total usage change, indicating also whether they undergo major isoform switching. **c** From left to right: gene, transcript, and protein-level expression charts for the *Cntn1* gene in the two conditions in our system (NPC and OPC, each containing two replicates—squares indicate replicate expression, points indicate mean replicate expression), and tappAS graphical representation of its transcript-level annotation. While there are no changes in the gene expression level (not DE), the gene presents differential isoform usage. Two alternative isoforms present the inclusion of a uORF due to an alternative TSS. **d** Multi-dimensional gene set enrichment analysis of genes ranked by DE and DIU significance level. Nodes correspond to GO terms obtained by selecting representatives of the top enriched GO term clusters. Pie chart area represents DE (blue) and DIU (orange) regulation measured by relative -log10(*p* value) of the enrichment test, and node size represents the number of genes annotated for each GO term

Figure 4a shows a summary of the results of differential module analyses (combined results for all analyses in Supplementary File 1) in the oligodendrocyte differentiation dataset. Gene expression regulation affected a larger number of genes than AltTEM (1205 genes were DE between NPCs and OPCs (FDR < 0.05, FC > 1.5) vs 538 genes significant for DIU; FDR < 0.05) (Fig. 4a). Interestingly, ~ 50% of DIU genes were not DE (Fig. 4a), indicating that a relevant fraction of the post-transcriptional regulation was independent of changes in the total gene expression. After applying a relatively low expression filter (examples in Figure S2A), we found 110 genes that were no longer DIU, indicating that lowly expressed isoforms may result in spurious DIU calls (Fig. 4a). Differential coding region usage (DCU) analysis showed that, for 279 genes, AltTEM led to changes in coding sequence usage, in contrast to 135 genes that were significantly DIU but not DCU (example in Figure S2B), and hence that most post-transcriptional regulation, although not all, may change protein properties. In addition, we observed that 35% of DCU genes showed isoform switching between NPCs and OPCs (Fig. 4a), identifying a set of genes where AltTEM regulation is likely to have strong functional impact.

In addition to switching, and to estimate more accurately the magnitude of the changes in isoform usage, we propose a new metric, *total isoform usage change*, i.e., the magnitude of redistribution of gene expression across its transcripts when conditions are compared. The fold change vs total isoform usage change plot displays the relative contribution of transcriptional and post-transcriptional effects to the regulation of gene expression (Fig. 4b). In the neural dataset, genes with a large total isoform usage change were likely to be significant for DIU and undergo isoform switching, regardless of gene expression fold change. This is the case of *Cntn1*, which encodes a cell adhesion protein and is key in neural development processes [77], including myelination [78], OPC development [79], and neuronal migration [80]. The isoform usage change in *Cntn1* caused the inclusion of a uORF via an alternative TSS in NPCs (Fig. 4c), which may exert a regulatory function in subsequent protein translation [25–27]. Our analysis also highlighted multi-isoform gene *Ctnnd1*, which encodes p120. p120 is a well-known component of the β-catenin signaling pathway, which has been shown to be an important process in the differentiation of NPCs towards OPCs [81, 82]. *Ctnnd1* encodes several transcript isoforms via the combination of two exon skipping events (Figure S3), which are regulated via AltTEM with no impact in the total gene expression (Fig. 7 b). In the murine neural data, we observed that most genes with isoform switching events had total usage change values > 20%, demonstrating the usefulness of these metrics as criteria to prioritize AltTEM-regulated genes for further experimental validation.

### Functional comparison of transcriptional and AltTEM regulation

To investigate the potential functional consequences of the identified transcriptional changes, tappAS implements a flexible framework for enrichment analysis where all available functional annotation categories, gene selections, and ranked gene lists can be combined. To illustrate these functionalities, we first ran a functional enrichment analysis of DIU and DCU genes using DE genes as comparing list. This allowed us to detect cellular processes and functionalities preferentially regulated by AltTEM, rather than by changes in the total gene expression levels. For both DIU and DCU genes, functions and components related to mRNA splicing were frequently identified as enriched (Figure S4A), while coding-related changes (i.e., DCU genes) were enriched in transcriptional and translational regulation terms, as well as synapse formation (Figure S4B). In addition, several RBPs, nuclear localization signals (NLS) and PTMs were among the most significantly enriched motifs among the AltTEM-regulated genes (Figure S4A and S4B).

In order to get a more comprehensive understanding of transcriptional and post-transcriptional regulation, our framework includes a Gene Ontology-based multi-dimensional gene set enrichment analysis [83]. Using this approach, DE and DIU genes ranked by significance can be combined to identify enriched functions controlled by either mechanism (Fig. 4d). In the murine neural data, processes active in neural differentiation were regulated in concert with changes in the total gene expression and in isoform relative abundance. AltTEM-regulated genes dominated in a core of GO terms related to neural morphogenesis (e.g., *neuron projection*, *axon part*, *neurogenesis*, *plasma membrane bounded cell projection*), differentiation (*cell differentiation*, *nervous system development*), and RNA metabolism (e.g., *regulation of RNA splicing*, *regulation of mRNA metabolic process*) (Fig. 4d) and were enriched in RNA-binding protein (RBP) sites (Figure S5). Genes from several splicing regulator families, such as Ser/Arg-rich splicing factors (*Srsf5*, *Srsf10*), Muscleblind-like proteins (*Mbnl1*, *Mbnl2*), and RNA-binding motif proteins (*Rbm5*, *Rmb7*) undergo significant differential isoform/protein usage in our system (Figure S5 and S12A). Interestingly, several aspects of vesicle transport also appeared to have preferential AltTEM regulation, including the interaction with the extracellular environment (e.g., *extracellular exosome*/*vesicle*), endocytosis (e.g., *receptor-mediated endocytosis*), the localization to intracellular compartments (e.g., *establishment of localization*, *cytoplasmic vesicle*, *endoplasmatic reticulum*), and some of their underlying molecular aspects (e.g., *vesicle uncoating*, *cellular response to calcium*) (Fig. 4d). This is in agreement with the known role of vesicle trafficking for polarity establishment and myelination [84–86]⁠, and with previous reports of splicing regulation of vesicle transport [87], also during differentiation [88].

In summary, these examples demonstrate that the differential module in tappAS readily enables the study the balance between differential gene expression and AltTEM in transcriptome analysis, while also illustrating their combined biological effects.

### Feature-level analysis of AltTEM

**Fig. 5:**
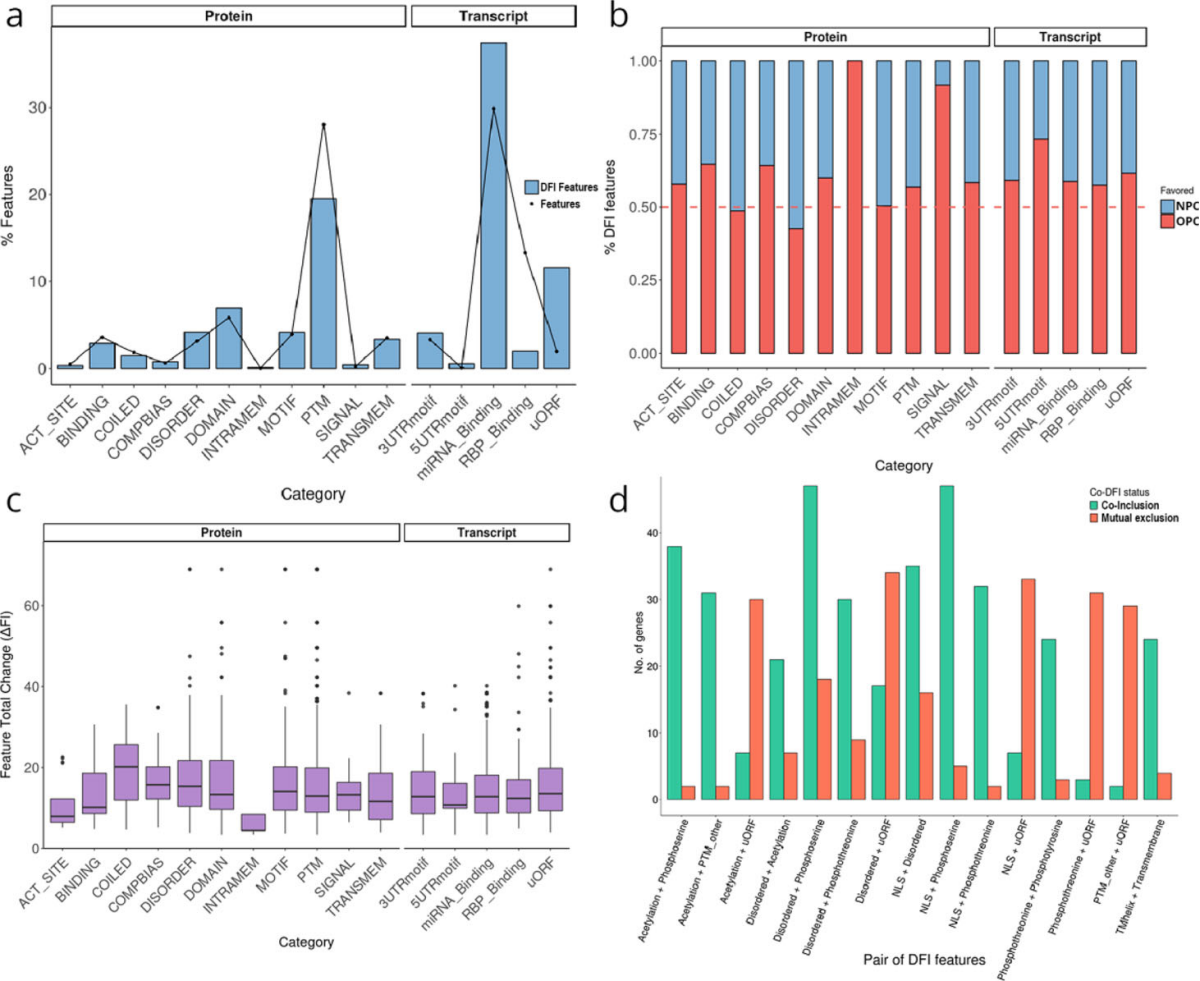
Summary of DFI results. **a** Distribution (%) of features annotated in the transcriptome (dots) vs differentially included features revealed by the analysis (bars). The relative over-representation of DFI features in specific categories is evaluated by Fisher exact tests and corrected for multiple testing using the Benjamini-Hochberg method. **b** Distribution (%) of differentially included features according to the cell type in which the inclusion of the feature is favored. Enrichment of the feature category in the cell type is calculated by a binomial test with probability = 0.5 and Benjamini-Hochberg multiple testing correction. **c** Barplot of feature inclusion differences (ΔFI) across feature category. For all DFI results, the significance of individual categories (see the “Methods” section) is marked by asterisks: ***p < 0.001; **p < 0.01; *p < 0.05. **d** Top 15 co-DFI associations ranked by the number of genes with both features marked as DFI. Bar color indicates the number of genes where features are co-included in the same conditions (co-inclusion) or in opposite conditions/groups (mutual exclusion)

Our previous functional enrichment analysis revealed an overrepresentation of certain motifs, signals, and domains in DIU and DCU genes, thus suggesting an interplay between the alternative isoform usage and the functionalities of different gene products (Figure S4). However, these analyses do not address the functional impact of AltTEM regulation, in particular, whether inclusion of specific functional elements is regulated by splicing in a condition-specific manner. To investigate how inclusion of functional features changes due to the differential usage of isoforms across conditions, we applied tappAS’ differential feature inclusion (DFI) analysis (Table 1, the “Methods” section). Significantly differentially included features between NPCs and OPCs were identified in 526 genes, including ~ 83% of previously detected DIU genes, indicating that post-transcriptional regulation modifies functional properties for the majority of genes. Features positive for DFI were found to be distributed across all functional categories (Fig. 5a, Table S6), although a significant relative enrichment was found for uORFs (Fisher’s exact test (FET) *p* value = 5.25e−121), RNA-binding protein (RBP) sites (FET *p* value = 2.46e−07), compositional bias regions (FET *p* value = 4.06e−03), and intrinsic disordered regions (IDRs, FET *p* value 5.02e−03), most of them also found significantly included when considering multiple feature occurrences only once per gene (Figure S6).

DFI analysis provides two novel metrics: the feature inclusion ratio—to identify the experimental condition where the feature is upregulated or *favored*—and the magnitude of the change (ΔFI, see the “Methods” section). Considering these metrics, we observed that feature gain tended to be more frequent in OPCs when compared to NPCs (Fig. 5b), which can be interpreted as AltTEM promoting the incorporation of functional properties as cells differentiate. For example, we observed OPC-specific inclusion of signal peptides (binomial test, probability of success = 0.5, BiTest FDR = 2.10e−02), as well as of miRNA binding sites (BiTest FDR = 3.85e−08), uORFs (BiTest FDR = 5.85e−31), and RBP binding sites (BiTest FDR = 4.09e−04), which may indicate a 3′ UTR lengthening trend in OPCs vs NPCs, a feature observed in mammalian brains [89]. Remarkably, when comparing (absolute) differences in feature inclusion rates between the two cell types, we found these to be around 20% for most categories, with some genes presenting features with ΔFI > 50% (Fig. 5c). In particular, coiled-coil regions were identified as the category with the highest inclusion changes between cell types (Fig. 5c). These results bring to light tappAS’ ability to provide functional category-level differential inclusion evaluations, as well as to capture the condition-dependent impact of AltTEM on individual functional elements.

Finally, there is a growing interest in understanding *cis*-associations across post-transcriptional events [90, 91]. We applied tappAS’ co-DFI analysis to calculate the coordinated inclusion of functional features in the murine neural data (Fig. 5d). Evaluation of the top occurring feature pairs revealed frequent associations between NLS and phosphoserine residues (examples in Figures S7A-B) and C2H2-type zinc finger domains (examples in Figures S7C-D). Interestingly, the post-translational masking of NLS is a known mechanism to prevent nuclear import [92, 93]⁠. IDRs are also co-included with phosphoserine residues, confirming their described role in the allocation of PTMs [94–96] (examples in Figure S7A-B).

### Differential polyadenylation

Alternative polyadenylation and differences at UTR lengths are involved in the regulation of mRNA stability, subcellular location, RNA protein binding, and translation efficiency [49, 97]⁠. To assess the contribution of AltTEM to these processes, we have developed novel differential polyadenylation (DPA) and 3′ and 5′ UTR Lengthening (3/5UL) analyses, again with new quantitative metrics (Table 1) (see the “Methods” section).

**Fig. 6:**
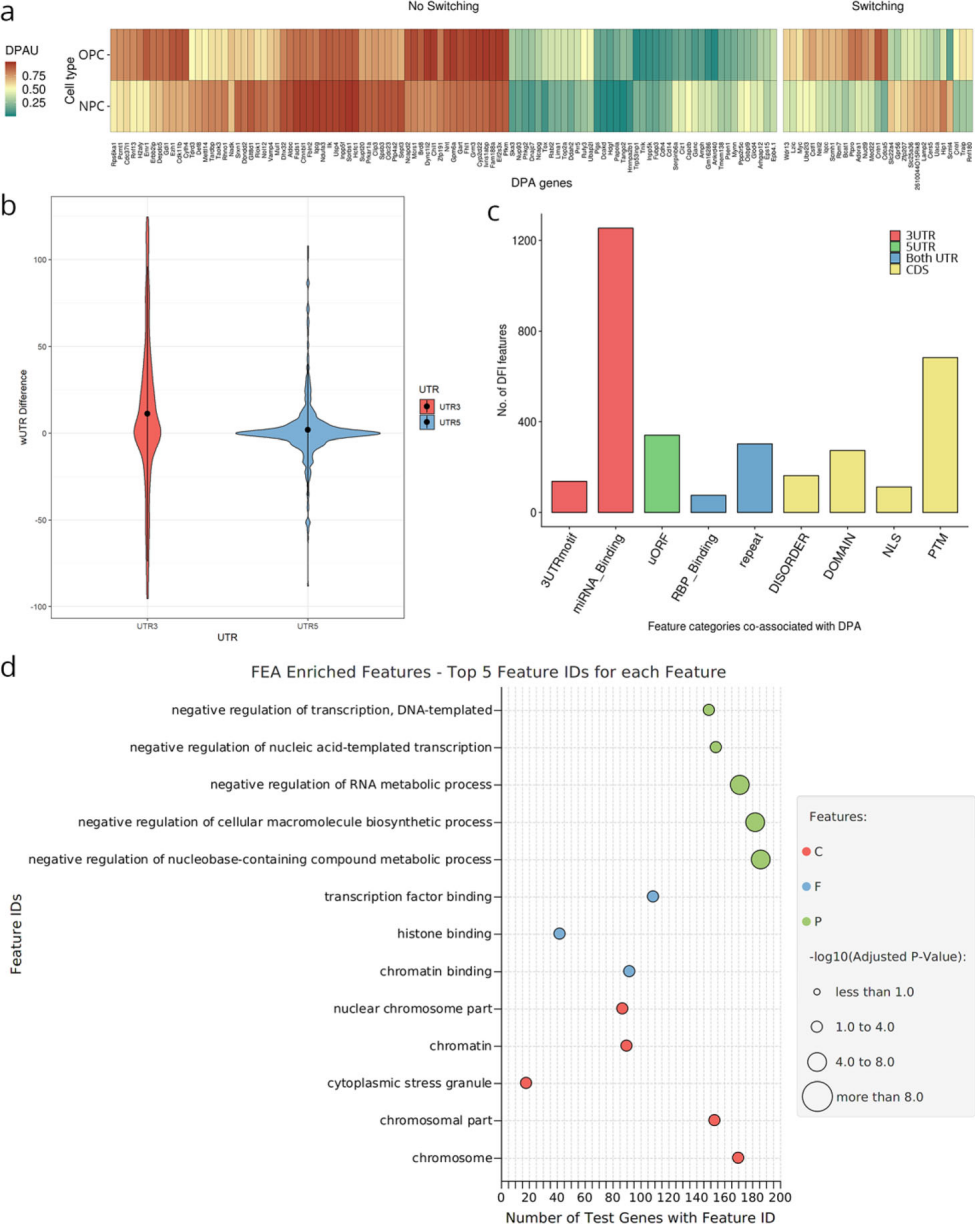
Differential polyadenylation analysis (DPA) results. **a** Heatmap displaying distal polyA usage (DPAU) levels of genes that are most significantly DPA (FDR < 0.001, threshold stringency increased for visualization purposes) for each cell type. **b** Violin plots showing the distribution of the difference in expression-weighted 3′ and 5′ UTR lengths (UTRw) between OPCs and NPCs. **c** Frequency of differential inclusion of features from several annotation categories for DPA (FDR > 0.05) genes. Bar color indicates the transcript region where the feature is present. **d** Functional enrichment of DPA genes for GO terms (Fisher’s exact test, multiple testing correction by Benjamini-Hochberg method, FDR < 0.05). Dot color indicates the GO term aspect, while dot size indicates significance level

Applied to the murine data, we found that 18% of genes with alternative polyA sites (see the “Methods” section) were positive for DPA (262 out of 1459, FDR < 0.05, minimum distance between distal and proximal sites, 60 bp), among which ~ 19% (50 genes) switched their major polyA site between cell types (Fig. 6a). Furthermore, consistent with the results showing the significant inclusion of 3′ UTR elements in OPCs (Fig. 4c), 3/5UL analysis results revealed a significant increase towards 3′ UTR lengthening for OPCs (Fig. 6b, Wilcoxon signed-rank test, *p* value = 2.267e−05, minimum distance to call different UTRs, 100 bp). Over 1200 motifs whose differential inclusion was associated with distal polyA site usage were detected, including 126 genes where APA regulation was associated to the presence of miRNA binding sites (Fig. 6c), further illustrating a potential combined UTR regulatory effect.

In agreement with FDA results (Table S5), miR-590-3p binding sites were the most frequently detected as DFI among APA-regulated genes (Figure S8A). Interestingly, also coding features, such as PFAM domains, showed co-occurrence with alternative polyadenylation (Fig. 6c). For example, C2H2-type zinc fingers were by far the most frequently DFI domain within the subset of APA-regulated genes (Figure S8B). This is a consequence of nearly all C2H2 domain annotations in our transcriptome being present in APA-regulated genes (90 of 91, 98.9%; outstandingly above APA-regulated/non-APA-regulated ratio, 18%) and suggests that UTR regulation might be an important component of C2H2 zinc finger proteins. An example of polyA site regulation affecting coding and non-coding features via a coding region APA (CR-APA) mechanism is *Rufy3*, a gene involved in the establishment of neural polarity [98]. *Rufy3* is affected by the APA-driven inclusion of miR-590-3p (Figure S9A) in the 3′ UTR, while simultaneously promoting the deletion of the C-terminal protein region at the protein level (Figure S9B). Notably, this *Rufy3* protein region has been reported to interact with RAB5A GTPase [99] as a part of a mechanism that controls intracellular membrane trafficking and co-localization in the large vesicle structures. This result, together with the fact that C-terminal lacking (and thus miRNA binding-containing) isoforms are favored in NPCs, indicates a coordinated and interdependent regulation of isoform-specific miRNA targeting and protein functionality.

Regarding broad biological functions affected by APA regulation, functional enrichment of DPA genes indicated that a significant number of these genes were involved in transcription regulation and RNA processing (Fig. 6d). One illustrative example is *Tdrd3*, a transcriptional activator in the nucleus that is also involved in the formation of stress granules and the regulation of mRNA translation in the cytoplasm [100]. Similar to *Rufy3*, *Tdrd3* undergoes CR-APA, resulting in an OPC-upregulated isoform with simultaneous inclusion of miRNAs binding sites and AU-rich elements (ARE) at the 3′ UTR, in agreement with associations detected between APA and differential inclusion of 3′ UTR features (Fig. 6c, Figure S10A). Moreover, the protein resulting from this UTR motif-containing isoform presents disruption of a phosphotyrosine site and an exon-junction (EJC) interacting region (Figure S10B), further stressing the coordination between the inclusion of CDS elements and APA. Altogether, these analyses illustrate the power of tappAS for combining UTR, polyA site, coding region, and domain tools to facilitate an in-depth analysis of the functional links associated to the alternative processing of transcript termination sites.

### Experimental validation of functional AltTEM events for tappAS candidates

**Fig. 7:**
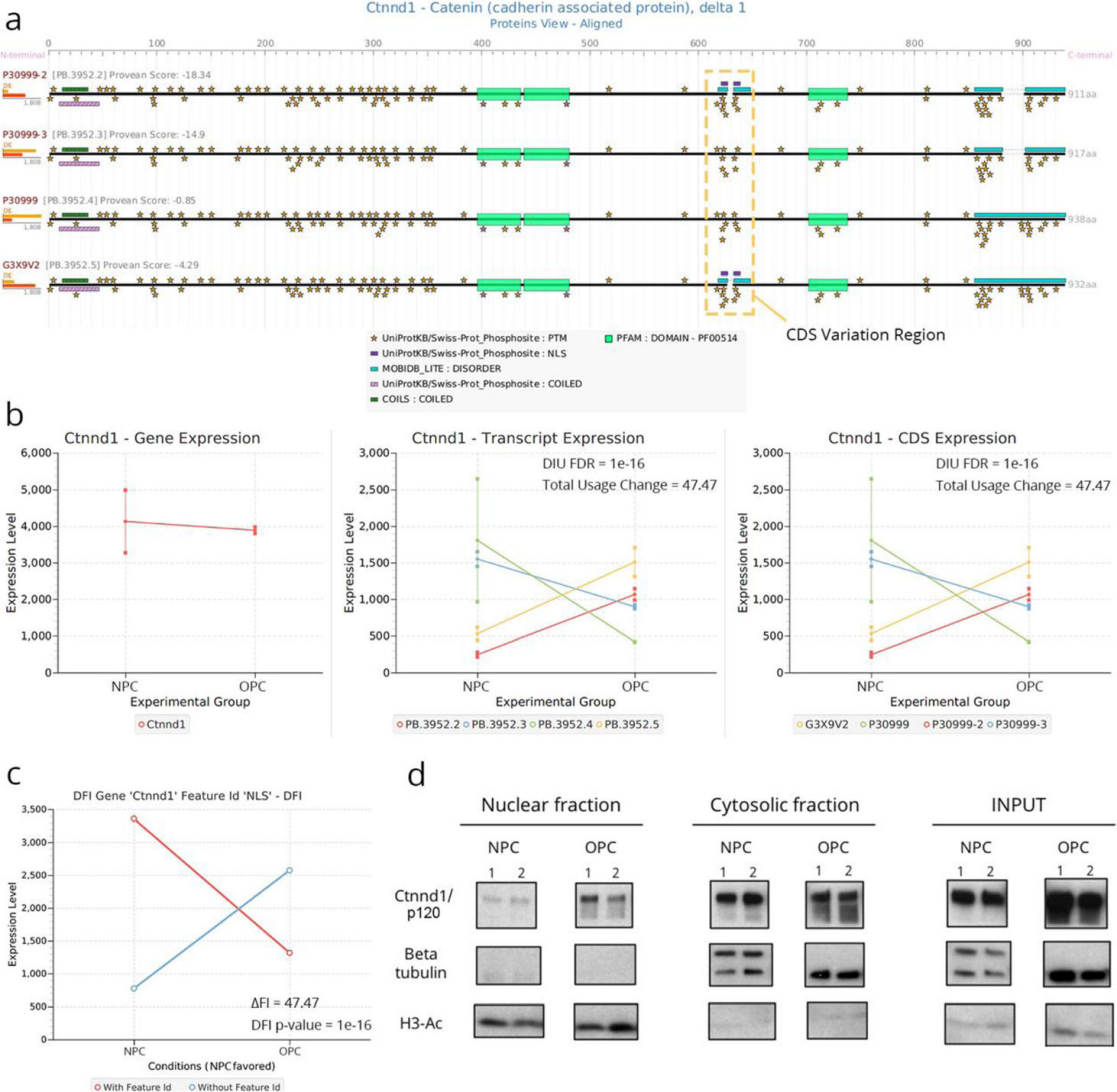
tappAS analysis results and experimental validation AltTEM processing of *Ctnnd1*/p120**. a** Protein-level visualization of tappAS functional annotation for *Ctnnd/p120*. Exclusion of an exon in two of its isoforms causes an NLS motif to appear in the protein sequence. **b** Gene-, transcript-, and CDS-level expression of *Ctnnd1*. The gene is significant for both DIU and DCU, with major isoform switching of the nuclear isoforms (yellow and red) in OPCs. **c** DFI analysis results for the NLS motif in *Ctnnd1*. NLS inclusion is favored in OPCs. **d** Western blot analysis of Ctnnd1 protein p120 in nuclear cytosolic, cytosolic, and total fractions of NPCs and OPCs. An increase of the nuclear expression of the protein is observed in OPCs due to differential inclusion of the NLS, while cytosolic expression remains constant

The tappAS tool implements a variety of analysis methods to study alternative transcript processing from a functional perspective. We examined the ability of the approach to generate valid functional hypothesis by experimental validation of two genes identified in our analyses. *Ctnnd1* (Fig. 3c, Figure S3C) shows the presence of a nuclear localization signal (NLS) in two alternative isoforms, which differ based on the inclusion of exon 10 (Fig. 7a). Total gene expression does not change between NPCs and OPCs, yet *Ctnnd1* is significant for DIU (FDR = 4.22e−8), having a large total isoform usage change (47.47) and clear isoform switching between cell types (Fig. 7b). NLS-containing protein isoforms are strongly downregulated in NPCs, resulting in significant differential inclusion of the NLS in OPCs (DFI FDR = 1e−16, Fig. 7c). We thus hypothesized that AltTEM regulation was responsible for a protein localization change in the Ctnnd1 protein (aka p120). Validation experiments using Western blot analysis of p120 in nuclear/cytoplasmic fractions for both cell types confirmed the increase of nuclear levels of the protein in OPCs in comparison to NPCs, where a cytoplasmic retention of p120 was observed (Fig. 7d). Densitometry quantification of Western blot bands indicated that the observed differences in nuclear/cytoplasmic protein abundance across cell types were statistically significant (Figure S11A, see the “Methods” section).

We detected several mRNA processing-related GO terms (Figure S4) and regulation of several splicing factors by AltTEM mechanisms (Figure S5) in our analyses. *Mbnl1*, an important neural development splicing factor [11] widely studied for its implications in neurological disorders [101–104], was unequivocally identified as AltTEM-regulated (DIU FDR = 0.0018, total change = 23.51, positive for isoform switching; Figures S12A-B). Similar to *Ctnnd1*, *Mbnl1* possesses a NLS that is differentially included (DFI FDR = 0.007) across differentially expressed isoforms. Nuclear presence of *Mbnl1* is favored in NPCs (Figure S12C). Western blot analysis also confirmed the predicted cellular localization pattern for the protein (Figure S12D), and densitometry quantification revealed a significant interaction between cell type and cellular localization (Figure S11B).

## Discussion

In this work, we present a novel analysis framework for the comprehensive functional analysis of isoform-resolved transcriptomes, referred here as Functional Iso-Transcriptomics (FIT). The overall concept behind FIT is that transcript variants of the same gene contain sequence differences in regions encoding functional elements. When modifying these sequences, either by the inclusion or the exclusion of exons or by lengthening or shortening of UTRs, transcripts gain or lose functional elements that, in turn, confer different functional properties. Therefore, the study of the functional impact of alternative isoform expression should be coupled to the study of the differential inclusion of these functional elements. This is in contrast with the traditional approach to the functional assessment of differential splicing, in which analyses are restricted to the functional enrichment of spliced genes. Comprehensive functional analyses however require both of an isoform-resolved annotation of a broad diversity of functional sites and novel computational methods to evaluate the abovementioned differences. We have implemented this concept, along with the traditional approach, in tappAS, a user-friendly software tool that includes novel metrics and analysis approaches for the functional analysis of AltTEM. We have included methods for the analysis of the variability in functional sites at genes with multiple transcript variants (FDA), strategies to evaluate the functional impact of the context-dependent expression of alternative isoforms (enrichment analyses of DIU and DCU genes), and ways to identify which functional elements change as a consequence of differential isoform usage (DFI). Each approach covers a different aspect of the analysis of the functional implications of AltTEM. FDA looks at the potential of a transcriptome to result in functional diversity by differential expression of transcript variants. In other words, it is only when different isoforms of the same gene incorporate different functional elements in their sequences that changes in their expression levels can have an AltTEM-mediated functional impact. While such an assessment depends on the comprehensiveness of the isoform-level functional annotation available in or provided to tappAS, which will vary from organism to organism, FDA helps evaluate these data and reports to what extent subsequent functional analyses of differential isoform usage may be meaningful. Moreover, tappAS identifies which functional processes are regulated by AltTEM both at the transcript level (by DIU analysis) and at the coding sequence level (DCU analysis). Notably, the latter is only useful when the actual changes in protein properties are of relevance. In cases where users are interested in non-translated regions, tappAS differential polyadenylation analysis (DAPA) and UTR lengthening/shortening may be used. Given that changes in the UTR length can be the result of both usage of alternative TSS and TTS, or driven by changes in exon inclusion altering the length of the coding sequence, we consider these two groups of analyses to be complementary. Finally, DFI, a novel analysis introduced in this work, reveals which functional domains are being regulated via changes in the expression of isoforms including them. Taken together, all of tappAS modules fulfill a dual role, since they explore both the biological processes and the functional features regulated by AltTEM. As a result, tappAS not only provides insights on the biology of post-transcriptional regulation, but also facilitates the design of validation experiments.

We have also incorporated different metrics to capture the most relevant AltTEM changes. This type of quantitative strategies, although well established for differential expression analyses, was not common in the methods for the study of alternative splicing. Hence, tappAS implements several options to eliminate minor transcripts from data—to avoid spurious differential isoform usage results—as well as measures of the magnitude of change in the relative expression of isoforms and switching event reports. These two metrics are available for all of the tappAS different modules and help identify the most pronounced changes in AltTEM regulation, i.e., those likely to have a stronger functional impact. The combination of all these functionalities makes tappAS a comprehensive solution for the functional analysis of transcriptomics changes at the isoform level.

tappAS constitutes a timely development as long-read technologies are becoming increasingly accessible, providing accurate identification of full-length transcripts. Quantification of isoforms currently relies on short reads, but it is expected that direct quantification of isoform expression will be within reach in the near future. The implementation of FIT in the tappAS framework is deliberately agnostic to the source of transcript models, sequencing platform, and species, and therefore can also leverage other recently proposed strategies to improve accuracy at transcript calls such as the combination of ChIP-Seq and RNA-Seq data [105] and the pre-filtering of reference isoforms based on event analysis [106].

While many methods to statistically evaluate the isoform expression differences exist, they either lack evaluation of full-length isoforms [37–40] or are limited to specific alternative processing events, such as APA [107–109], intron retention [110, 111], or alternative TSS [112–114]. Furthermore, methods aiming to provide functional interpretation to isoform usage are either limited to coding features [41, 43, 54] or rely on the user-collected annotations [55]. Therefore, an integral approach to test the function associated with isoform differences was missing. The tappAS software is designed to be flexible, enabling a diverse set of functional analysis of isoforms. At present, tappAS includes pre-computed annotation files for humans, mice, flies, *Arabidopsis*, and maize, together with auxiliary functions—IsoAnnot_Lite_—to map these annotations to long-read data or user-defined transcriptomes of the same species. We have demonstrated the potential of our functional approach to the analysis of AltTEM on a murine neural system, successfully recapitulating much of the existing knowledge about splicing and UTR-level regulation and generating a novel functional hypothesis that was experimentally validated.

The premise of FIT has been flexibly implemented with options for specifying gene sets or combining multiple functional layers, thus enabling scientists to interrogate the data in ways that are tailored to their own biological questions. Video tutorials at the tappAS website (https://app.tappas.org) guide users through several scenarios enabling complete customization of hypotheses to be tested. Annotation files can be directly uploaded by users so that any species and annotation source can be analyzed. For example, protein-protein interactions, lengths of polyAs, or conservation scores are not included in the current precomputed annotation data. Users with reliable annotations of these information layers can include this information and immediately test their differential inclusion across transcript variants. Similarly, as the tool is not limited by organism, but only by the current availability of annotation, other species not yet supported in the application will benefit from tappAS as functional information becomes available.

## Methods

### Retrieving isoform-resolved functional annotation features

tappAS uses a gff3-like file with transcript structural and functional data. To retrieve functional annotation features at both RNA and protein levels, we use available databases and state-of-the-art prediction algorithms (Table S1). Features are gathered through two mechanisms: positional transfer from functional databases and de novo prediction by state-of-the-art algorithms for sequence-based prediction. All functional labels annotated at the isoform resolution are positionally described via their exact localization within protein/RNA molecules.

For the present study, we generated an annotation file for a de novo murine transcriptome [62] (Table S3). RNA-level annotations included *cis*-acting UTR regulatory elements and upstream open reading frames (uORFs) predicted by UTRscan [115], repeat regions and low-complexity elements predicted by repeatMasker [116], and miRNA binding sites collected from mirWalk2.0 [117]⁠. A minimum seed length of 7 bp and a *p* value threshold of 0.05 were set as requirements to call miRNA binding sites. We filtered the site list by the number of sources reporting the association. Specifically, we kept miRNA binding sites predicted by at least 5 different methods, among which Targetscan [118], miRanda [119], and mirWalk [117] were required, mirWalk providing transcript coordinate information to locate miRNA binding sites. High-confidence miRNAs were identified using the experimental evidence information in miRbase [120], to include only those entries with the following experimental evidence: cloned, Northern, PCR, RT-PCR, qRT-PCR, 5’RACE, RTPCR, in-situ, qPCR, miRAP cloned, 3’RACE, insitu, RACE, miRAP, primer-extension, and RAKE. This strategy returns a total of 511 miRNAs with annotated binding sites and experimental evidence. Binding sites for 20 RNA-binding proteins (RBPs) were annotated by collecting genomic crosslinking immunoprecipitation (CLIP) data from CLIPdb [121] and mapping sites to isoforms.

At the protein level, Pfam domains were mapped with InterProScan [122]⁠, transmembrane regions predicted with TMHMM [123]⁠, signal peptides obtained by SignalP 4.0 [124], coiled-coil regions predicted by COILS [125]⁠, single and bipartite nuclear localization signals mapped by cNLS mapper [126]⁠ (score > 6), and disordered regions obtained by MobiDB Lite [127], which derives consensus IDR predictions by combining 8 different predictors. We predicted isoforms containing a premature termination codon (PTC)—potentially leading to non-sense-mediated decay (NMD)—using the 50-nt rule [128]⁠ that indicates that a termination codon situated more than 50–55 nt upstream of an exon-exon junction is generally a PTC.

In addition to sequence-based prediction methods, some protein-centric databases contain a detailed annotation of protein features. However, these are generally biased towards the annotation of the best-documented isoform, hindering the study of the functional diversity of alternative isoforms. To correct this, we map canonical isoform annotations to query isoform sequences, novel or known, following an isoform-aware positional transfer strategy. We obtained the information on protein functional features by parsing UniprotKB [129] and PhosphoSitePlus [130] databases. In both cases, we deal with the disparities between databases when defining gene models and ensure the ORF and genomic position conservation between public and query sequences during feature transference. As a result, we retrieved an extensive set of post-translational modification (PTM) sites with experimental evidence from PhosphoSitePlus and a diverse catalog of functional sequence features from UniprotKB.

Pre-computed gff3 files with isoform functional data for mouse, human, Arabidopsis, fly and maize reference transcriptomes have also been generated. Specific details can be found in Tables S1 and S2. Alternatively, custom gff3 files for long-read sequencing (LRS) data can be obtained for these species by running the IsoAnnot_Lite_ algorithm, available at https://isoannot.tappas.org in the form of a python3 script. Note that annotation transference requires LRS data to be previously curated using SQANTI [62]. IsoAnnotLite takes *_corrected.gtf, *_classification.txt, and *_junctions.txt SQANTI output files, together with the pre-computed gff3 of the species of interest, as input, and returns a new gff3 file where tappAS annotations have been transferred to the LRS transcripts, including novel transcript variants of existing genes. At present, IsoAnnot_Lite_ does not annotate transcripts in novel genes in these species; however, these typically account for 1–2% of the LRS output.

### Visualization engine of positional functional annotation at isoform resolution

The tappAS visualization engine is designed to display isoform variability in a user-friendly manner. Using the visualization power of the Java engine, tappAS displays the whole catalog of isoform-resolved annotation features and their position using a distinctive icon on both transcript and protein isoform structure maps. Maps include UTR/CDS areas, polyA sites, splice junction and exon information, and functional features, creating a graphical representation that greatly facilitates the study and comparison of isoform diversity.

### Functional diversity analysis

Isoforms vary in structural and functional features among isoforms of the same gene. FDA identifies and measures the nature of the variability in a qualitative manner. For every annotation category, all pairwise comparisons between transcript isoforms from the same gene are performed to evaluate whether at least one isoform pair has variability in a feature, either in its annotated genomic position(s) (*positional varying*) or in the presence/absence of the annotated feature (*presence varying*). Functional diversity can be assessed by gene or by feature ID.

#### Gene-level diversity

The gene-level diversity analysis evaluates genes as a function of the structural, functional, and regulatory features that are modulated by AltTEM and reports the diversity rate for each category. Depending on the category and its relationship to the functional properties of a transcript or protein, functional diversity is evaluated using a *positional varying* strategy or a *presence varying* strategy.

The *positional varying* approach compares features by genomic position, i.e., by mapping features to genomic coordinates and classifying them as varying if coordinates are not equivalent between gene isoforms. Position disagreement is annotated when > 9 bp, that is, 3 amino acids, allowing for variability in prediction*. Presence varying* includes only the presence/absence of annotation. For instance, NMD transcript status is based on differences in the transcript-level NMD label. In contrast, transcript attributes such as UTR length, CDS, and polyA site positions are examples of features where only *positional* evaluation is meaningful. However, the third group of features (such as Pfam domains or transmembrane regions) can be affected by AltTEM via both complete skipping and partial disruption of the feature. In these and similar cases, both strategies can be used and provide complementary insight on AltTEM in the potential regulation of the functional or regulatory feature.

For structural features evaluated by *positional varying*, some special cases arise. In order to detect alternative polyadenylation (APA) events, polyA sites are identified as the last genomic position of transcript isoforms and evaluated in a pairwise manner by computing the polyA distance between each pairwise combination of isoforms expressed by a given gene. mRNA cleavage is not an exact process and can occur within a small window of positions [131]. To take cleavage variability into account, a pair of isoforms is labeled as APA when there is a minimum *X* bp genomic distance (default value 100) between polyA sites. In order to analyze UTR variability, UTR length is computed for each isoform for a subsequent pairwise comparison between coding isoforms from the same gene. Pairs of isoforms with 3′/5′ UTR differences above a user-specified cutoff (75 bp by default) are labeled as 3′/5′ UTR length varying, respectively. Finally, CDS variability is determined by comparing CDSs both at the sequence and genomic coordinate levels. Non-coding isoforms are discarded from CDS diversity analysis.

#### Feature-level diversity

The feature-level diversity analysis identifies specific functional and regulatory elements (i.e., by feature ID instead of source/functional category) varying across isoforms from the same gene. The diversity status of each ID can also be evaluated via *positional* and *presence varying* or both. Feature-level diversity analysis evaluates global rates of variation for the feature IDs contained in a given category and tests their enrichment in varying status using Fisher’s exact test [132]. Significance values are then corrected using the Benjamini-Hochberg [133] method for multiple testing correction.

### Differential feature inclusion analysis

DFI applies the concept of exon inclusion analysis to functional features. DFI is only applied to features labeled as varying—either by position or as present/absent—across gene isoforms, as only these have the potential to be significantly regulated. For a given gene and functional element, the null hypothesis that transcripts containing the feature have equivalent expression to transcripts not containing the feature is tested for each gene. Expression values of the isoforms containing the feature, and isoforms where the feature is partially or completely absent, are calculated from the data.

The *feature inclusion rate* is defined as the ratio between the sum of the expression of all feature-including isoforms and the total expression of the gene (i.e., the sum of the expression of isoforms including and excluding the feature) for each condition studied:

$$ {\mathrm{FI}}_{fg}=\frac{{\mathrm{EInc}}_{fg}}{{\mathrm{EInc}}_{fg}+{\mathrm{EExc}}_{fg}} $$

where EInc is the aggregated expression value for feature-including isoforms, and EExc is the aggregated expression value for feature-excluding isoforms for gene *g* and positional feature *f*.

Differential inclusion of functional features is then tested using DEXSeq [38]⁠ and maSigPro [134] methods, for two-group and time-course experimental designs, respectively. Each gene-feature pair is individually examined. For each model, the significance of the condition-variant or condition-variant-time interactions is evaluated, depending on the experimental design considered. When multiple functional annotation categories are analyzed (domains, UTR motifs, disordered regions, etc.), each of them is tested and *p* values for each test are corrected by FDR separately. The FDR and the significance threshold for each comparison are set to 0.05 by default.

### Co-differential feature inclusion analysis

co-DFI analysis evaluates the co-occurrence of significant DFI for two features for the same gene in the same condition, while mutual exclusion evaluates how often two features are simultaneously DFI for the same gene in different conditions. co-DFI is computed for each pair of features detected as DFI in at least 5 genes.

### Defining a library of polyA sites

tappAS uses a polyA site database that is created by extracting the genomic coordinate of the last position of each transcript isoform. Unlike recently developed tools [109], polyA sites in terminal exons with different 5′ start sites are also considered to allow the analysis of alternative polyadenylation sites affecting either coding (CR-APAs) or UTR (UTR-APAs) events. Non-coding isoforms as well as NMD-predicted variants are discarded.

Next, a series of filtering and collapsing steps are performed in order to define the proximal (pPA) and distal polyA (dPA) sites for each gene. First, independent cleavage sites are defined by merging polyA sites located within a 75-bp window. To avoid the definition of a minor polyA site as a distal or proximal site, a filter based on relative polyA site expression levels is applied and only polyA sites accumulating at least 10% (default threshold) of total gene expression in at least one condition are considered. In the case of genes with more than two polyA sites, we perform a final merge of unlabeled sites by assigning them to the nearest proximal or distal site.

### Differential polyadenylation analysis

Using the defined polyA site library, tappAS computes the per-gene and per-sample dPA and pPA site expression levels by collapsing the expression levels of the set of transcript isoforms that contain either the dPA or the pPA. Condition-variant interactions are tested with either DEXSeq or maSigPro as above. The relative distal polyA site usage (DPAU) is calculated as the relative expression of the sum of all isoforms containing the distal site over the total polyA site expression level of the gene:

$$ \mathrm{DPAU}=\frac{{\mathrm{E}}_{\mathrm{dPA}}}{{\mathrm{E}}_{\mathrm{dPA}}+{\mathrm{E}}_{\mathrm{pPA}}} $$

where EdPA and EpPA correspond to the expression levels of the variants defined as distal and proximal polyA sites, respectively.

### Detecting lengthening and shortening of 3′ UTRs

For isoforms with identical CDS end positions but different polyA (UTR-APAs) distal/proximal polyA site usage, UTR lengthening/shortening events are implicated. However, when changes in polyA site position imply changes in the CDS (CR-APAs), it is impossible to directly infer the relationship between the polyA site and 3′ UTR length. Since DPA analysis assesses polyA site regulation independently of the coding sequence, a specific 3′ UTR lengthening/shortening analysis that computes an isoform usage-weighted UTR length for each condition is used:

$$ {\mathrm{UTR}}_w=\sum \limits_{i=1}^n{U}_{ig}\cdotp {\mathrm{UTR}}_{ig} $$

where *U* is the relative usage of isoform *i* in gene *g*, and *UTR* is the associated 3′ UTR length.

UTRs from highly expressed isoforms will contribute in a higher proportion to the final UTR mean length. The weighted UTRs are a measure of the actual extent of UTR length changes across conditions. Statistical differences are tested by using a Wilcoxon rank-sum test of the weighed UTR values.

### tappAS software

tappAS (http://tappas.org) is a Java GUI application that implements this broad analytical framework and includes a range of functions that, collectively and in combination, allow the study of different structural and functional aspects associated to isoform usage. Statistical methods are implemented in R and are run in the Rscript environment. See http://tappas.org for a comprehensive list of R package dependencies, as well as other software and hardware requirements.

tappAS is developed in modules and works using projects, each of them created using two inputs: a transcript expression matrix and an experimental design file, which can either be a two-group comparison or a time-course experiment. Being a GUI application, a rich set of interactive features are available in tappAS via the JavaFX platform, including customizable data tables, complex sorting and filtering options, data and figure export, context-sensitive help pages, data drill-down, and display customization. Increasing the flexibility of the tappAS software, user-defined gene lists are allowable as input for analysis.

The structural annotation included in the gff3 is used to sum the transcript expression given as input for an estimate of the expression of genes. Similarly, CDSs are calculated as the sum of transcripts having the same ORF. Differential expression analyses can then be run at each of these aggregation levels.

tappAS implements existing tools when appropriate, including NOISeq [67] and maSigPro [134] for differential gene expression and DEXseq [38] and Iso-maSigPro [135]⁠ for differential isoform usage. The latest two methods assess DIU by fitting generalized linear models (GLMs) and testing the significance of the isoform-condition interaction coefficient, as proposed in [136]. Implemented enrichment methods are GOSeq [64] (functional enrichment), GOglm [65] (gene set enrichment), and mdgsa [83] for multi-dimensional GSEA. These enrichment tools can be easily applied to the results of any of the statistical methods included in tappAS. Finally, tappAS implements extant complementary functionalities (i.e., low-count expression filtering, TMM normalization [63], PCA, clustering methods) that enable the pre-processing and flexible exploration of data and results. Further details are available in the User Guide.

### Complementary metrics for isoform analysis

#### Major and minor isoforms

In tappAS, the major isoform of a gene is defined for each condition under study as the isoform with the highest expression, whereas the remaining isoforms of are labeled minor forms. However, in multiple time-course series, the major isoform is defined for each experimental group individually as the one with the highest mean expression across time points. This is to provide a reference point and does not assume functional relevance.

#### Isoform pre-filtering

For genes that express multiple isoforms, frequently, only a few isoforms are responsible for the major proportion of gene expression [137], while the rest may be present at low expression levels. tappAS allows for low expression filtering upon data upload; however, still, some isoforms may remain relatively minor in expression across conditions. When the minor isoforms have small expression changes between conditions, but these occur in the opposite direction to the predominant isoforms, significant isoform condition coefficients may be observed. To avoid the detection of DIU genes because of the “flat” behavior of minor isoforms, an isoform filtering step should be applied before statistical modeling. Two filtering approaches are implemented in tappAS. One considers the proportion of a gene’s expression represented by each isoform and filters those under a minimum expression rate (10% by default), while the other calculates the fold change of the minor isoforms vs the major and those below a specified fold change (FC) threshold (default FC = 2) are removed. Filtering options are enabled by the user.

#### Total usage change

##### Total usage change in DIU analysis

The fold change is a measure of magnitude in differential expression. In DIU analysis, where multiple isoforms are tested in a single model, we propose a new metric, *total usage change* to quantify the magnitude of change for DIU genes. *Total usage change* measures the amount of redistribution (as %) in the expression levels across different conditions for isoforms of the same gene. Because absolute gene expression levels may be different across conditions, total change values are represented as a function of the gene expression FC.

We define isoform usage as the relative expression of isoform *i* in gene *g*. Then, total usage change can be defined as:

$$ \sum \limits_{i=1}^n\left|\overset{\mathrm{IsoformUsageC}1}{\overbrace{\frac{E_{1 ig}}{\sum_{i=1}^n{E}_{1 ig}}}}-\overset{\mathrm{IsoformUsageC}2}{\overbrace{\frac{E_{2 ig}}{\sum_{i=1}^n{E}_{2 ig}}}}\right|\times 100\times 0.5 $$

where *E*_*ig*_ is the expression value for isoform *i* and gene *g*, and *n* represents the total number of isoforms for gene *g.*

##### Defining total usage change for feature analyses

When performing DFI and DPA analyses, the expression values are collapsed to obtain feature inclusion (FI) and distal polyadenylation site usage (DPAU) levels. In this case, total change is defined as the redistribution (as %) of FI (ΔFI) or DPAU (ΔDPAU) levels across every pair of conditions considered.

#### Defining switching events

*Switching events* identify differential feature inclusion, differential polyadenylation, and differential isoform usage events that change direction between conditions and can be used to prioritize candidates for further analysis.

A *major isoform switching event* occurs when the major isoform in one condition becomes a minor isoform in the other condition/time point. In multiple time-course series, major isoforms are defined for each experimental group. A major switch occurs when this isoform differs between groups. *Feature switching* (in DFI) and *distal polyA usage switching* (in DPA) are similarly defined.

##### Favored conditions

In DPA and DFI analyses, switching information is complemented by the information of the favored condition, i.e., the experimental condition where the inclusion of the feature is greatest.

### Experimental setup in murine neural cells

As a proof-of-concept of our analysis framework, we used the data from Tardaguila et al. [62]. Briefly, this dataset includes two different cell mouse cell types generated by in vitro differentiation of mouse spinal cord primary cells: Neural precursor cells (NPCs) and oligodendrocyte progenitor cells (OPCs) (two replicates per cell type). cDNA libraries were generated using the SMARTer kit (Takara Bio) and divided for sequencing with Illumina (60M reads) and PacBio RSII (0.6M reads). Transcriptomes were defined using long reads and the Iso-Seq PacBio pipeline and curated using SQANTI [62], resulting in 11,970 transcripts from 7167 genes. We next computed isoform expression levels of the SQANTI-filtered transcripts with Illumina reads using RSEM [66] following ENCODE guidelines.

### Validation of events with potential functional impact

#### Nuclear/cytoplasm isolations

Pellets of 5 million NPCs and OPCs were lysed in 1 ml of Farnham Lysis Buffer (FB, Pipes 5 mM pH = 8, KCl 85 mM, IGEPAL-670 0.5%, and a tablet of cOmplete Protease Inhibitor Cocktail, final volume 40 ml). Prior to sonication, aliquots of 41.6 μl were taken, mixed with Sample Buffer (SB, 4.8 μl of 4× NuPAGE LDS Sample Buffer and 1.92 μl of 10× NuPAGE Sample Reducing Agent per 10.8 μl of lysate) and boiled as an input control. The samples were transferred to 1-ml COVARIS MilliTUBES and kept on ice until sonicated in an S220 COVARIS sonicator for 5 min, peak power 75 W, 200 bursts/cycle, and 2% duty factor. Next, the samples were centrifuged at 1000*g* 4C for 5 min, and the supernatant was kept as the cytoplasm (Cyt) fraction. The pellet was subjected to four cycles of washing with 1 ml of FB resuspension and centrifugation at 1000*g* 4C for 5 min and finally resuspended in 50 to 100 μl of FB depending on its size. This constituted the nuclear fraction (Nu). If debris was observed in the wall of tubes after the initial separating centrifugation, the Cyt fraction was re-centrifuged. Aliquots of 20.8 μl of the Cyt and Nu fractions were resuspended in SB, boiled, and stored at − 80 °C.

#### Western blot

Input, Nu, and Cyt aliquots of three independent experiments were thawed, loaded completely in BioRad pre-cast 4–20% Mini-PROTEAN TGX Stain-Free Protein Gels (10 wells), and run at 150 V constant. Transference to a 0.45-μm nitrocellulose membrane (BioRad) was done at 300 mA constant for 1 h 30′ in a cold room in 20% methanol transfer buffer. The membrane was blocked with 4% milk in TBS with 0.5% Tween-20 for 1 h at room temperature and incubated at 4 °C overnight with the following primary antibody solutions (4% milk, 0.5% Tween-TBS): anti-p120 (Ctnnd1) 1:2000 (Millipore 05-1567, clone 15D2); anti-Mbnl1 1:100 (DSHB-MB2a(3b4)), and anti-Ac H3 1:1000 (Millipore 06-599). The membranes were incubated for 1 h at room temperature with secondary antibodies in 4% milk, 0.5% Tween-TBS: anti-mouse HRP (Life A16072) 1:10,000; anti-rabbit HRP (Thermo 31460) 1:10,000. Signal detection was performed with an enhanced chemiluminescence kit (ECL Plus Western blotting detection reagent from GE Healthcare, Piscataway Township, NJ, USA), and bands were detected by film exposition. After the first of detections, the membranes were quenched in 0.05% sodium azide in 0.5% Tween-TBS for 30 min with gentle rocking, washed three times with 0.5% Tween-TBS, and hybridized with anti-tubulin coupled HRP (Thermo MA5-16308-HRP) to produce cytoplasmic loading controls.

#### Densitometry analysis of the Western blot bands

ImageJ software was used for quantitative comparisons between bands from Western blot analysis. Equivalent rectangular areas were selected for each band, and the numerical intensity of each band was subsequently measured as the area under the curve of the histograms representing the brightness of the image. Densitometry data for each fraction (cytosolic, nuclear, and input) and cell type (NPCs and OPCs) was normalized by dividing the target protein expression (either p120 or Mbnl1) by the densitometry-measured expression of the relevant Western blot controls (H3-Ac for nuclear fractions, beta-tubulin for cytosolic and input fractions). The significance of the observed localization changes was evaluated by fitting a linear model (lm() function in base R) comparing the localization (aka cytoplasmic or nuclear isoforms) ~ cell type terms. When significant (*p* value < 0.05), the interaction between the two factors indicates that protein localization is significantly changing between cell types, and therefore, that AltTEM is the driver of the localization change.

## Supplementary information

**Additional file 1.** Supplementary figures and tables. This PDF file contains all of the supplementary figures and tables for this paper.

**Additional file 2.** This .tsv file contains combined results containing DE, DIU and isoform switching results for genes, transcripts and proteins (where applicable), as output by tappAS.

**Additional file 3.** Review history.

## Data Availability

Sequencing data used in this study was previously published by Tardaguila et al. [62] and submitted to the NCBI Sequence Read Archive (SRA; https://www.ncbi.nlm.nih.gov/sra) under study accession number SRP101446. The functional annotation file from the long read-defined mouse neural transcriptome used for the present analyses can be obtained at https://app.tappas.org/resources/downloads/gffs (file: Mus_musculus_Demo.zip). The tappAS application can be downloaded at https://app.tappas.org/downloads. tappAS source and compiled codes corresponding to this manuscript’s version of the software (v1.0.0) are available at https://github.com/ConesaLab/tappAS [138], under open source license GPL-3.0.
