# Supplementary material for: tappAS: a comprehensive computational framework for the analysis of the functional impact of differential splicing
Source: Genome Biol. 2020 May 18;21:119. doi: 10.1186/s13059-020-02028-w (PMC7236505; doi:10.1186/s13059-020-02028-w)
Supplement: Supplementary file 1 — Additional file 1. Supplementary figures and tables. This PDF file contains all of the supplementary figures and tables for this paper. [file 13059_2020_2028_MOESM1_ESM.pdf]

Additional File 1: Supplementary Figures and Tables

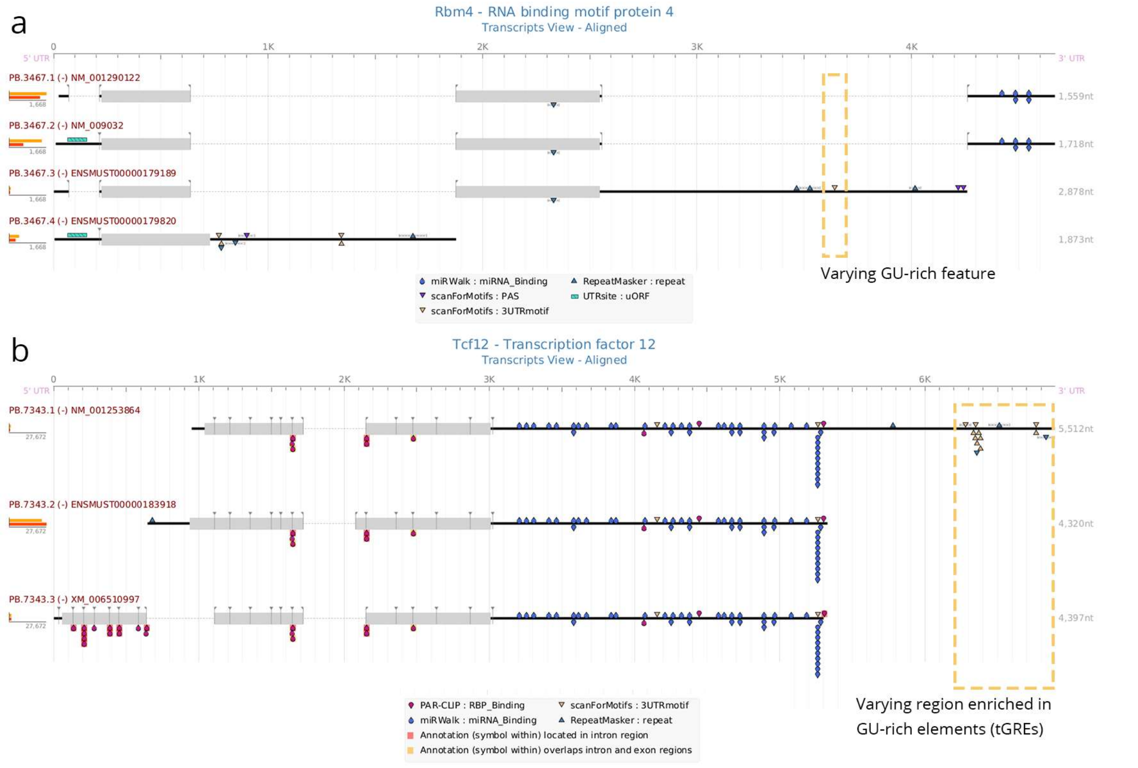

Figure S1: tappAS visualization of functional feature variation across isoforms. A) The *Rbm4* gene presents transcript-level variation in the inclusion of a GU-rich element (GRE) in the 3'UTR due to an exon-skipping event. B) Transcript-level variation in one of the isoforms of the *Tcf12* gene, which includes a 3'UTR region enriched in GREs due to an alternative TTS.

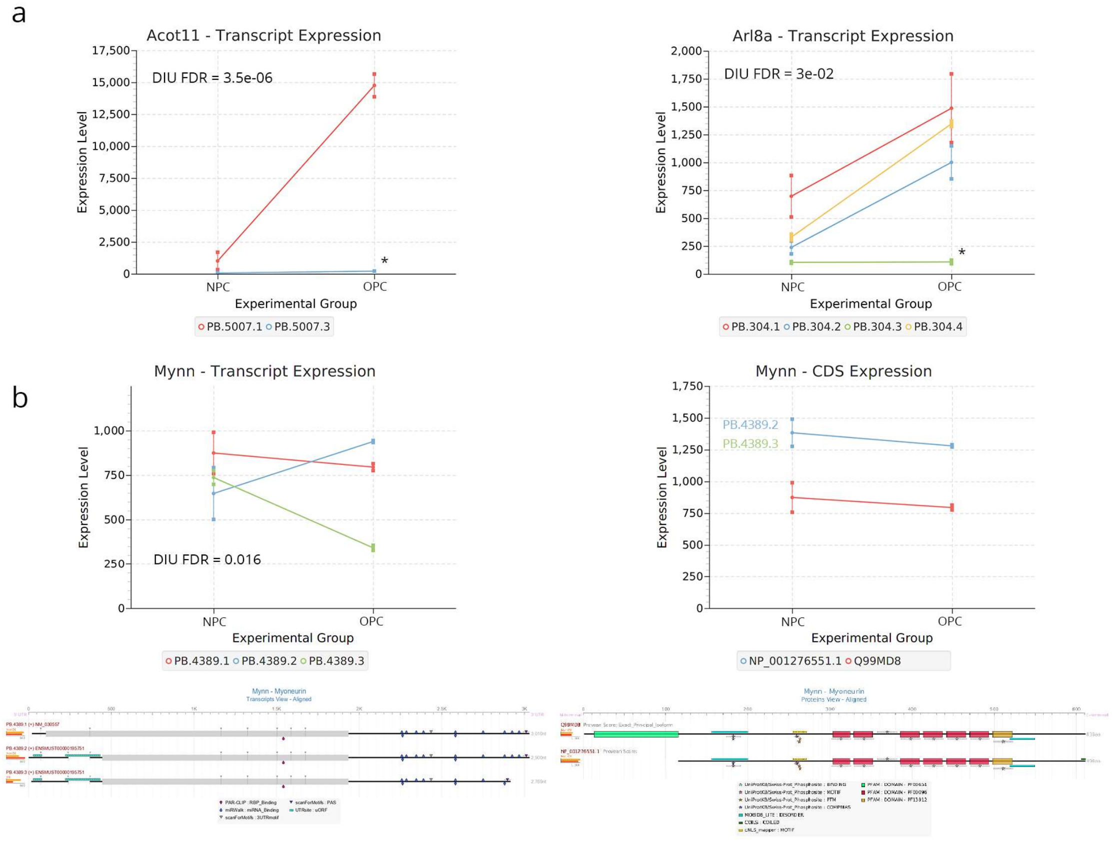

Figure S2: DE and DIU analysis results. A) Two examples of genes (*Acot11* and *Arl8*) detected as false positives for Differential Isoform Usage after minor isoform filtering (% expression < 0.1), i.e. where removal of the minor isoform leads to no DIU status. (*) indicates transcripts that were removed after minor isoform filtering. B) Expression charts and tappAS visualization of annotated functional features at the transcript (left) and protein (right) levels for the *Mynn* gene, where Differential Isoform Usage and major isoform switching imply no Differential Coding sequence Usage. Transcripts encoding the same protein are indicated using the PacBio transcript labels.

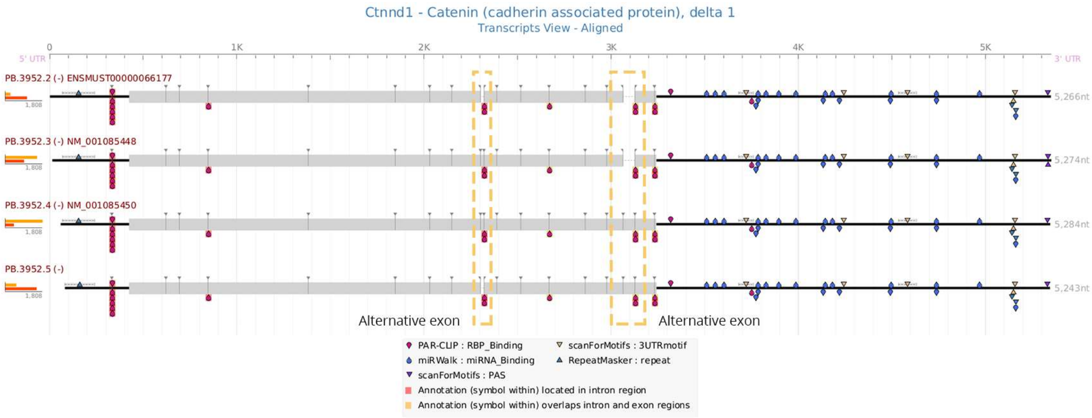

Figure S3: tappAS transcript view of the *Ctnnd1* gene. AltTEM events (i.e. alternative exons) that are different across isoforms are highlighted where relevant.

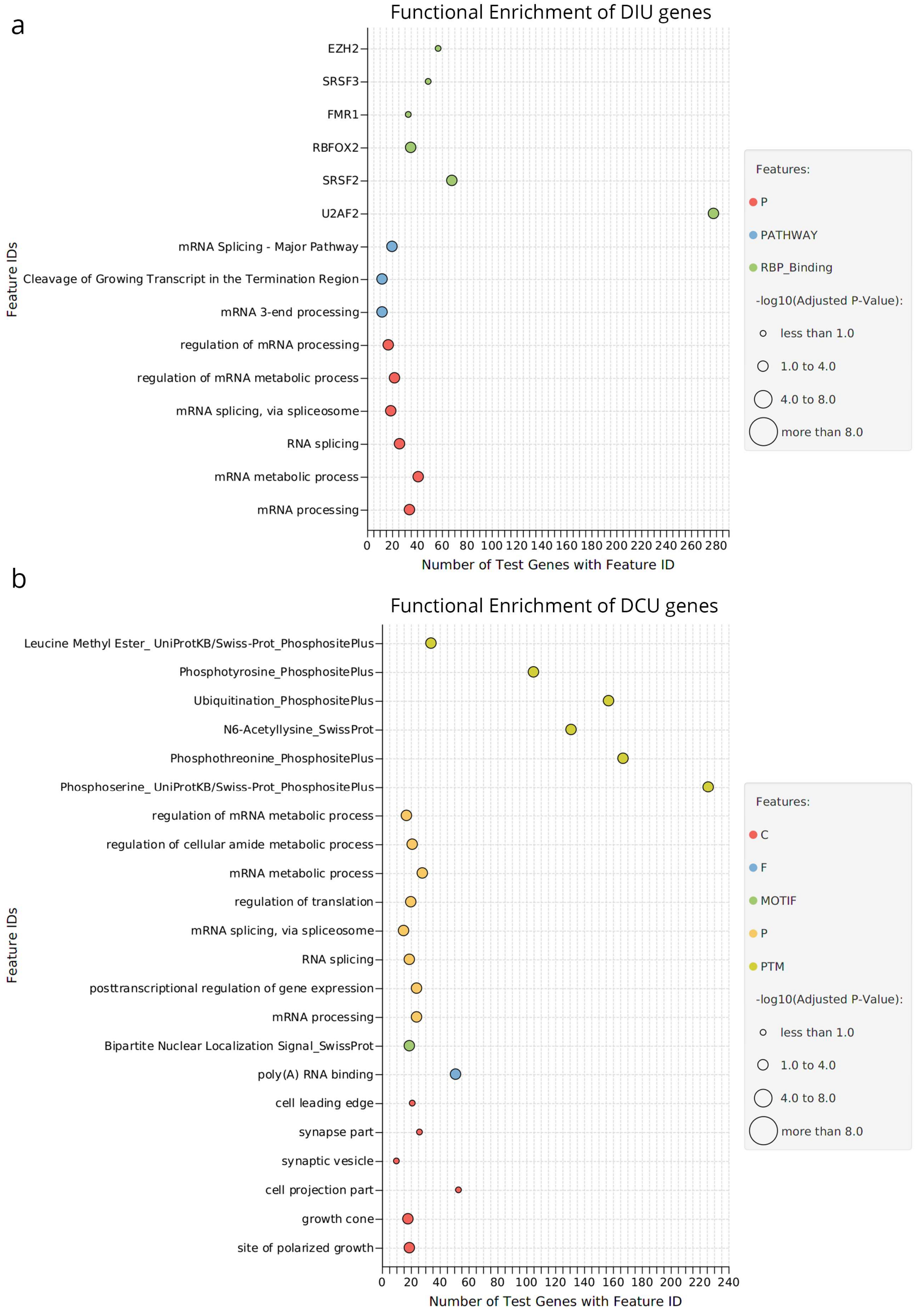

Figure S4: Functional Enrichment Results (Fisher´s Exact Test, with Benjamini-Hochberg multiple testing correction) for DIU (A) and DCU (B) genes (minor isoform filtering: proportion < 10%) using DE genes as background. Dot color indicates the functional category of the feature, while dot size indicates significance.

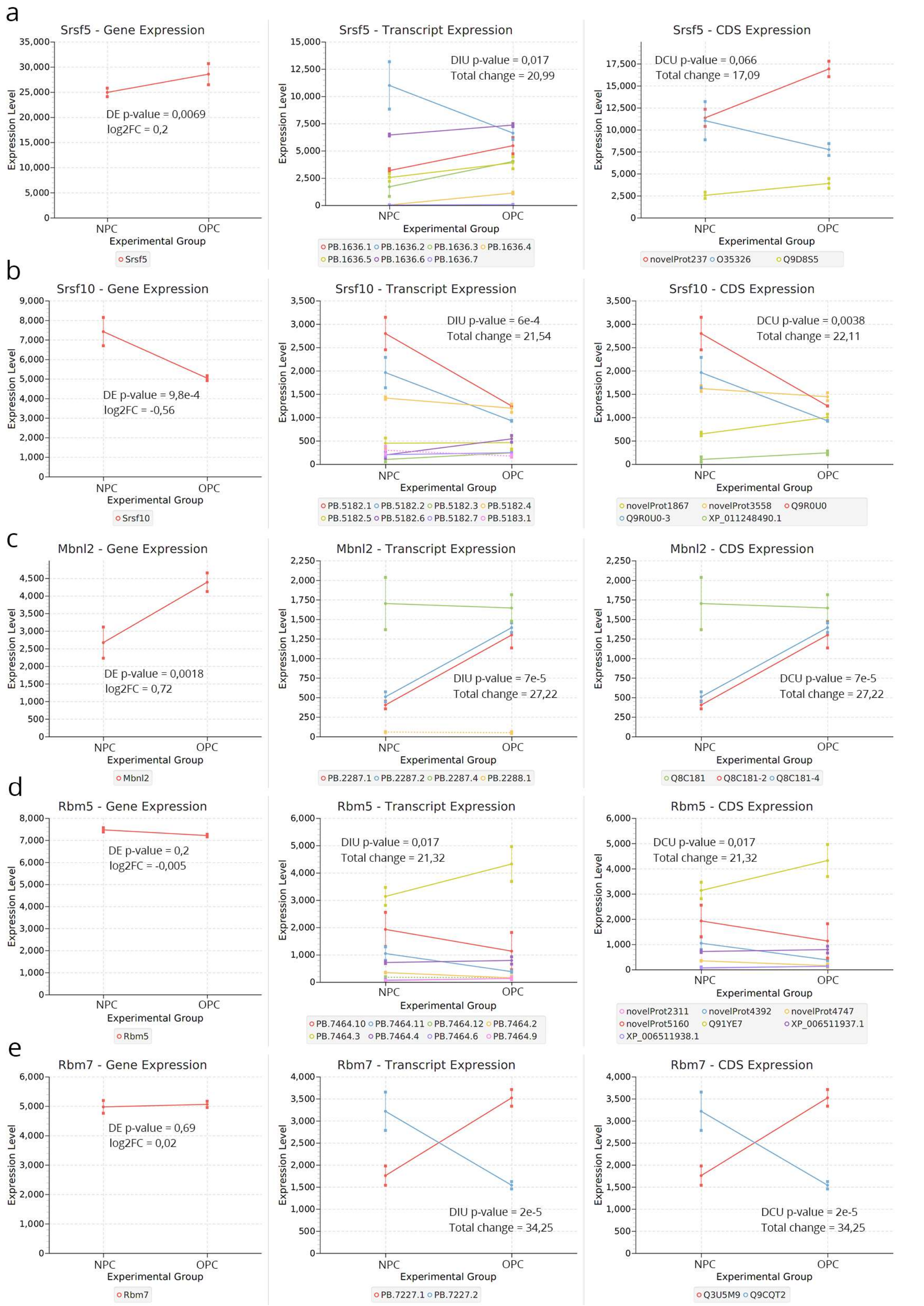

Figure S5: Splicing factors regulated by DIU. Transcript, gene and protein expression levels providing evidence of DIU status and self-regulation of the AltTEM machinery: A) *Srsf5*, B) *Srsf10*, C) *Mbnl2*, D) *Rbm5*, E) *Rbm7*. DIU and/or DCU (indicated only when significantly different from DIU results) significance corresponds to multiple testing adjusted FDRs.

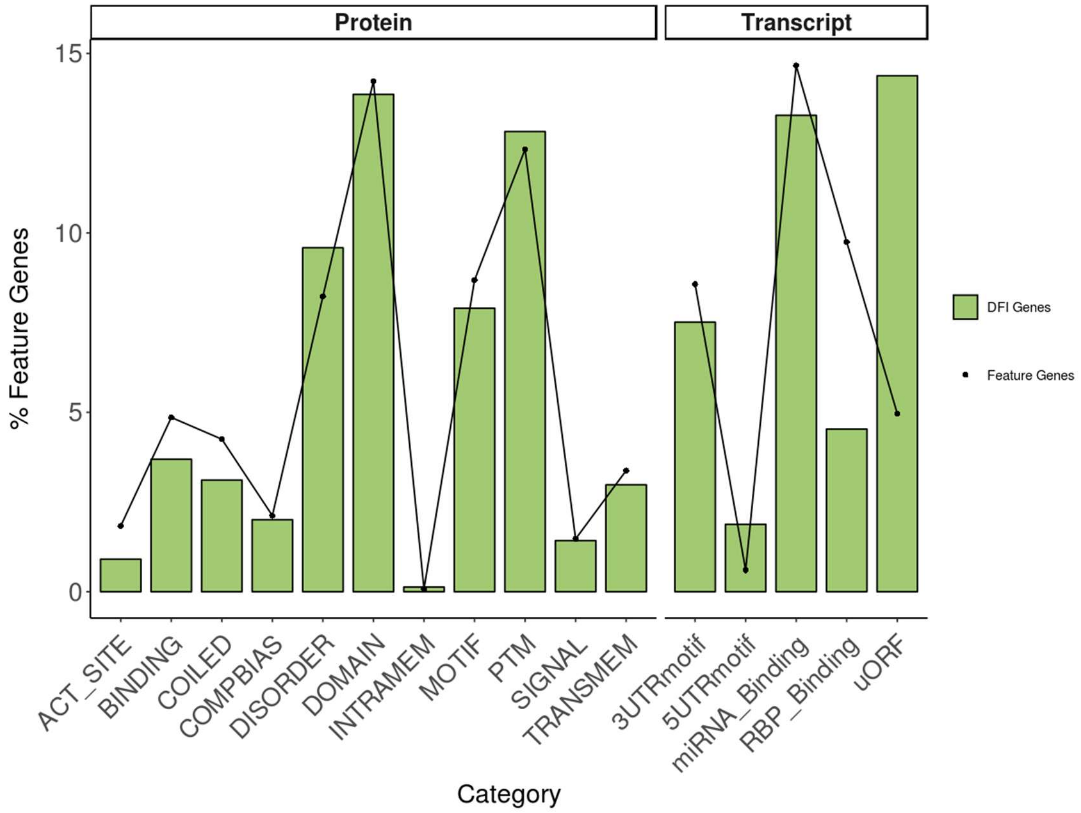

Figure S6: DFI results by gene. Distribution (%) of features annotated in the transcriptome (dots) vs differentially included features revealed by the analysis (bars). The relative over-representation of DFI features in specific categories is evaluated by Fisher Exact tests and corrected for multiple testing using the Benjamini-Hochberg method. Multiple occurrences of features belonging to the same annotation category in a single gene were considered only once. Significant categories are marked by asterisks: (***) $p < 0.001$; (**) $p < 0.01$; (*) $p < 0.05$.

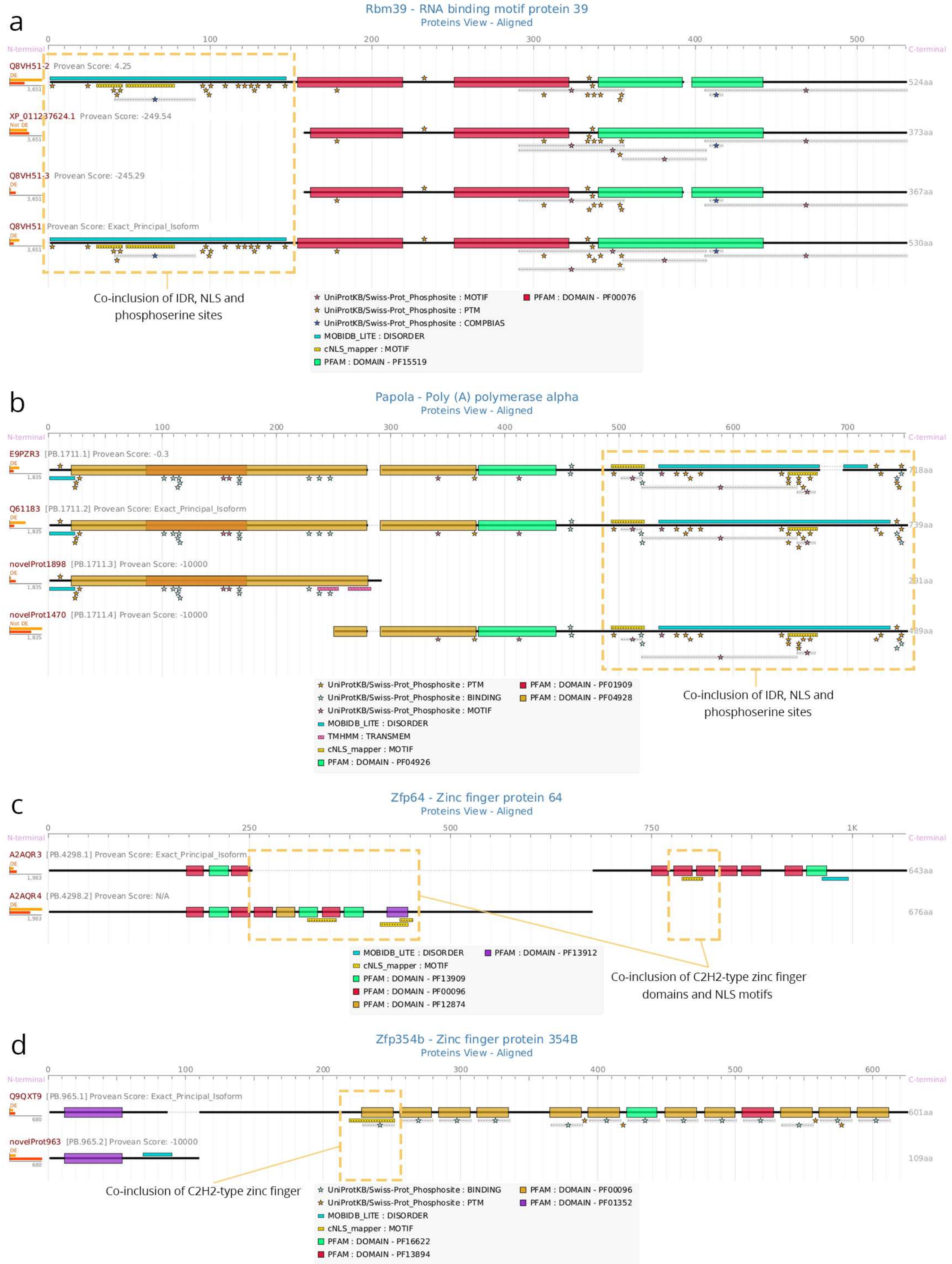

Figure S7: co-DFI results examples. A) Variation in the inclusion of protein-level functional elements in the *Rbm39* gene, which presents co-DFI status for an Intrinsically Disordered Region (IDR, DISORDER), several phosphoserine residues (PTM) and a Nuclear Localization Signal (NLS, MOTIF). B) Protein-level functional elements in the *Papola* gene, which presents co-DFI status for an IDR (DISORDER), several phosphoserine residues and two NLS (MOTIF). C) Protein visualization of he *Zfp64* gene, which presents co-DFI status of several C2H2-type zinc finger domains (PF13912, PF00096 and PF13909) and NLS motifs. D) The *Zpf354b* gene presents co-DFI status of a C2H2-type zinc finger domain (PF00096) and an NLS motif.

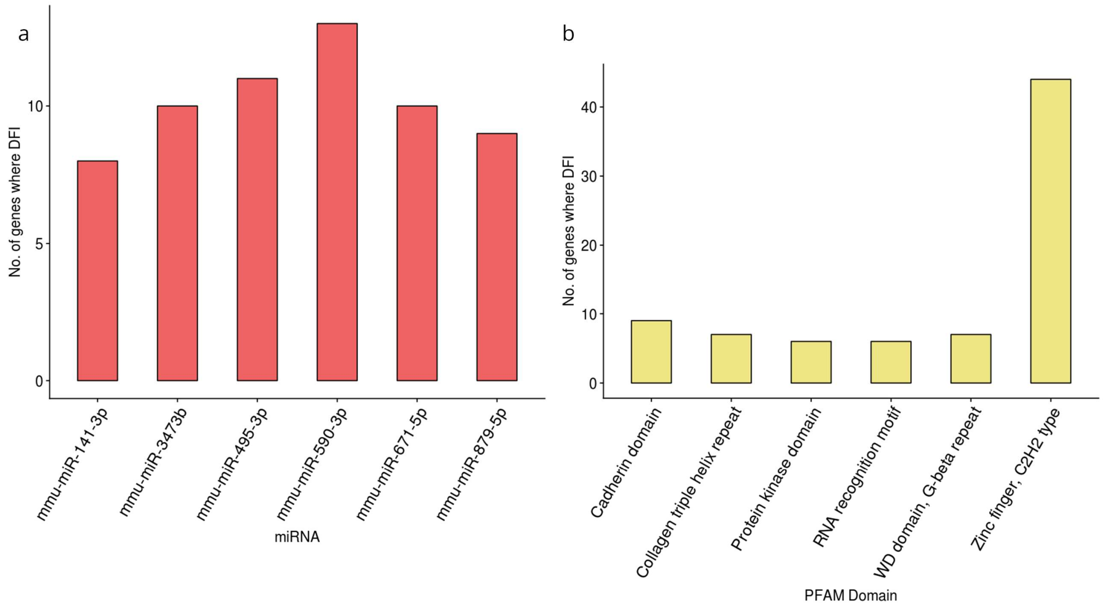

Figure S8: co-occurrence at the gene level between DPA and DFI for several feature categories. DFI frequencies (i.e. number of genes that are simultaneously DFI for a given feature and significant for DPA) are shown for A) top-6 most frequently DFI miRNA binding sites among DPA genes and B) top-6 most frequently DFI PFAM domains among DPA genes.

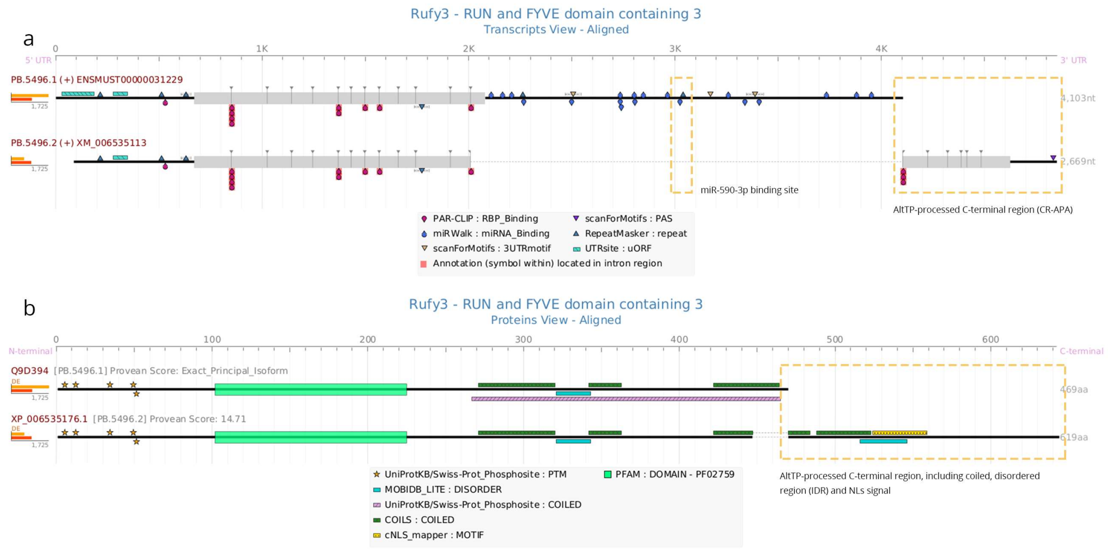

Figure S9: tappAS visualization of the *Rufy3* gene. A) tappAS visualization of transcript-level annotation for the *Rufy3* gene, where Coding Region -APA induced inclusion of miR-590-3p binding site is coupled with disruption of the C-terminal part of the protein. B) *Rufy3* protein-level annotation, showing missing C-terminal coiled, disordered regions (IDRs) and NLS signal.

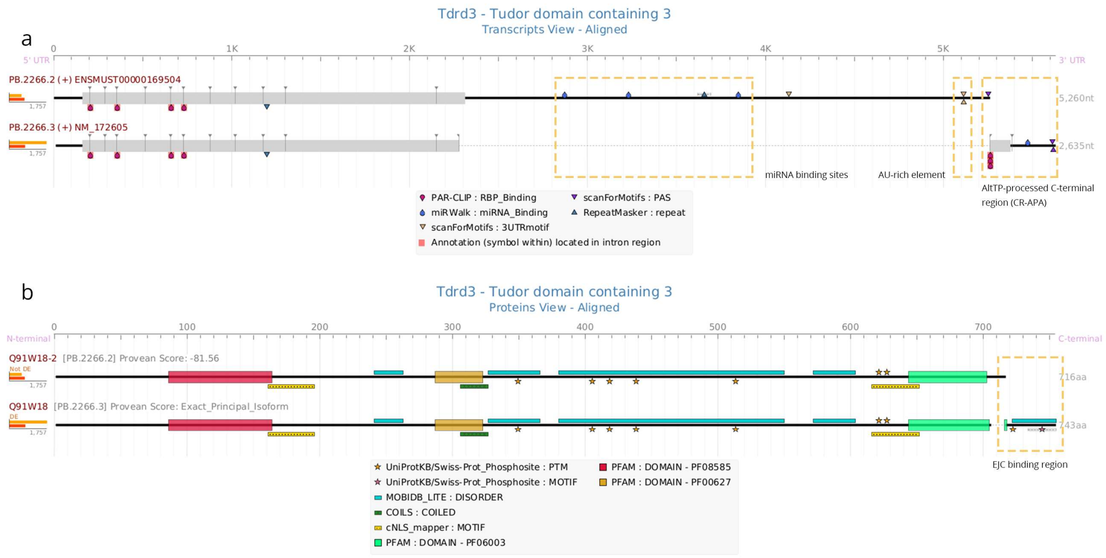

Figure S10: tappAS visualization of the *Tdrd3* gene. A) tappAS visualization of transcript-level annotation for the *Tdrd3* gene, where Codign Region -APA induced inclusion of several miRNA binding sites as well as an AU-rich element can be observed. B) *Tdrd3* protein-level annotation, where an EJC binding motif variation is caused by CR-APA-driven differential processing of the C-terminal region.

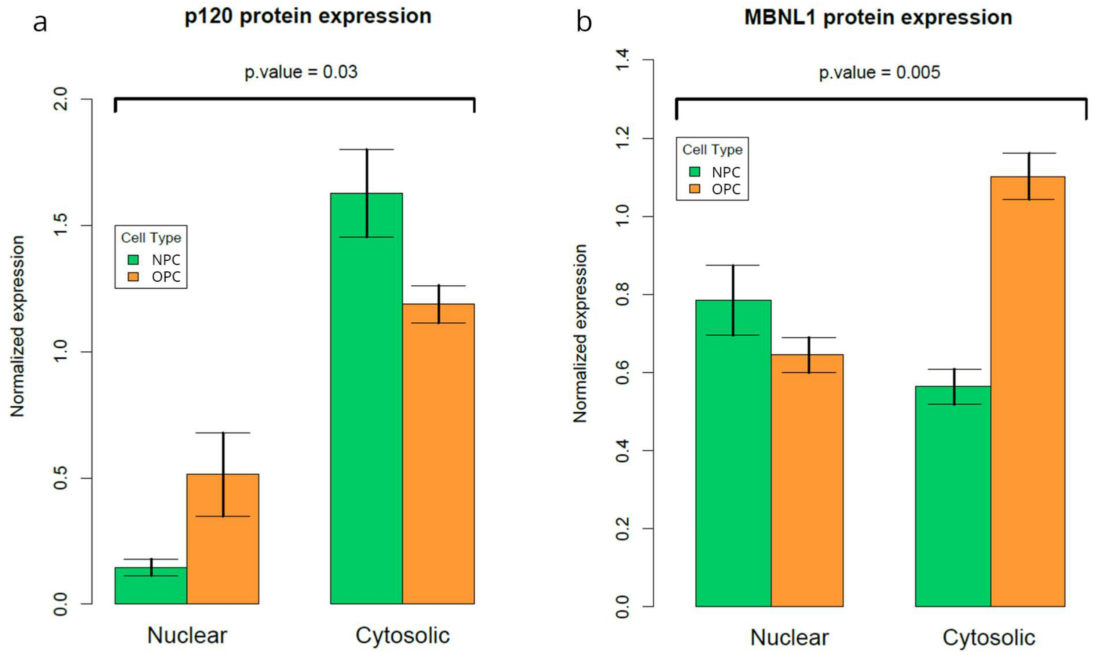

Figure S11: Western blot densitometry results for A) p120 (Ctnnd1) and B) Mbnl1. For each cell type (NPCs and OPCs), protein expression values obtained in densitometry after normalization with the relevant Western blot controls (H3 for nuclear, tubulin for cytosolic) are shown for nuclear and cytosolic fractions. Error bars correspond to standard deviations obtained after replicate 1 and 2 quantifications. P-values obtained by linear model fitting (see Methods).

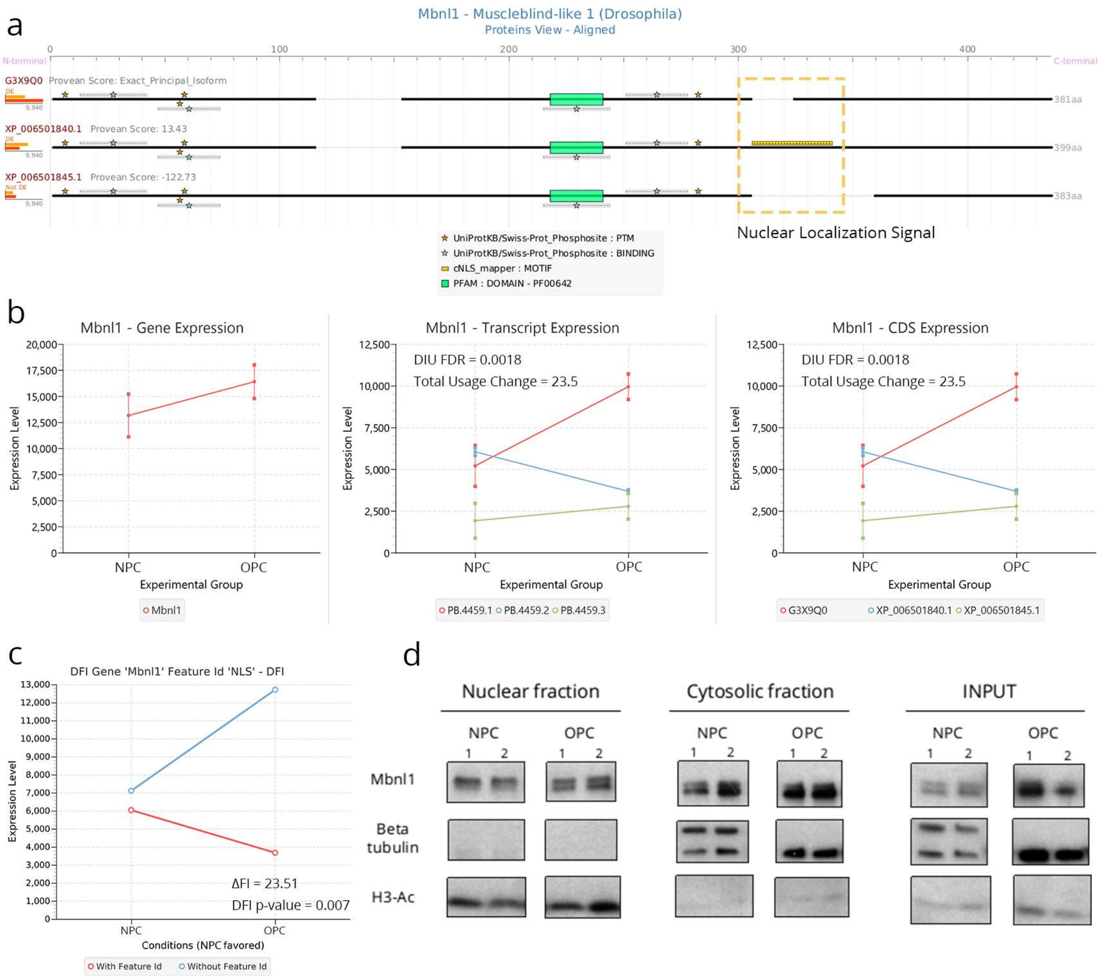

Figure S12: ***Mbnl1* AltTEM results.** A) Gene, transcript and CDS expression for *Mbnl1*. The gene is positive for DIU both at the transcript and protein level. B) tappAS visualization of *Mbnl1* functional annotation. Differential inclusion of an NLS signal is detected by tappAS comprehensive annotation. C) DFI results for *Mbnl1* NLS signal. The feature is significantly differentially included, and its inclusion favored in NPCs. D) Western blot analysis of *Mbnl1* in cytosolic and nuclear fractions of NPCs and OPCs. Together with a general increase in *Mbnl1* expression in OPCs (INPUT), an increase in protein levels in the cytoplasm is observed, likely due to exclusion of the NLS signal (Cytosolic fraction).

ISOFORM-RESOLVED ANNOTATION CATEGORY	SOURCE/PREDICTOR	ORGANISM	REFERENCES
		TRANSCRIPT LEVEL	
Nonsense-mediated decay (NMD)	In-house scripts	mouse/human/fruit fly/Arabidopsis thaliana/maize	Zhang et al., 2009
miRNA	miRBase	mouse/human	Kozomara et al., 2019
miRNA binding sites	miRWalk2.0	mouse/human	Dweep et al., 2015
miRNA binding sites	DIANA/microT	fruit fly	Paraskevopoulou et al., 2013
miRNA binding sites	DORINA/Pictar2	fruit fly	Anders et al., 2012
miRNA binding sites	miRaNda	fruit fly	Betel et al., 2010
miRNA binding sites	PITA	fruit fly	Kertesz et al., 2007
miRNA binding sites	RNA22v2	fruit fly	Loher & Rigoutsos, 2012
miRNA binding sites	TargetScan	fruit fly	Friedman et al., 2009
miRNA binding sites	psRNATarget	Arabidopsis thaliana/maize	Dai et al., 2018
PolyAdenylation Signal	UTRscan/UTRdb	mouse/human/fruit fly/Arabidopsis thaliana/maize	Grillo et al., 2010
Upstream open reading frames	UTRscan/UTRdb	mouse/human/fruit fly/Arabidopsis thaliana/maize	Grillo et al., 2010
3' UTR regulatory elements (AU-rich, GU-rich...)	ScanForMotifs	mouse	Biswas et al., 2014
5'-UTR and 3'-UTR regularotory elements	UTRscan/UTRdb	mouse/human/fruit fly/Arabidopsis thaliana/maize	Grillo et al., 2010
Repeat regions and low complexity elements	RepBase RepeatMasker	mouse/human/fruit fly/Arabidopsis thaliana/maize	http://www.repeatmasker.org
RBP binding sites	CLIPdb	mouse	Yang et al., 2015
		PROTEIN LEVEL	
PFAM domains	PFAM-HMMER3	mouse/human/fruit fly/Arabidopsis thaliana/maize	Finn et al., 2014
SMART domains	SMART-HMMER3	Arabidopsis thaliana/maize	Letunic et al., 2017
TIGRFAM protein families	TIGRFAM-HMMER3	Arabidopsis thaliana/maize	Haft et al., 2001
classifical nuclear localization signals	NLSmapper	mouse	Kosugi *et al* ., 2009
Signal Peptides	SIGNALP 4.0	mouse/human/Arabidopsis thaliana/maize	Petersen et al., 2011
Coiled-coil regions	COILS	mouse/human/Arabidopsis thaliana/maize	Lupas et al., 1991
Disordered Regions	MobiDB Lite	mouse	Necci et al., 2017
Transmembrane regions	TMHMM Server v. 2.0	mouse/human/Arabidopsis thaliana/maize	Krogh et al., 2001
Protein motifs, sites and regions	UniprotKB	mouse/human/fruit fly/Arabidopsis thaliana/maize	The UniProt Consortium, 2019
Post-translational modifications	PhosphositePlus + UniprotKB	mouse/human/fruit fly/Arabidopsis thaliana/maize	Hornbeck et al., 2012

Table S1: Resources used to generate the isoform-resolved functional annotation files available in the tappAS application.

Organism	Reference	# Isoform Resolved Functional Features	Mean Features/ Isoform	% Isoforms with Functional Features		
				Transcript Level	Protein Level	Total
Mus musculus	PacBio (Tardaguila et al., 2018)	386,114	35.23	98.08	98.20	99.73
	RefSeq78	2,628,525	23.61	90.24	96.38	94.66
	Ensembl86	1,314,089	11.32	59.17	92.70	68.11
Homo sapiens	RefSeq78	5,977,941	38.48	94.42	98.19	96.40
	Ensembl86	2,888,409	15.31	65.03	95.38	71.88
Drosophila melanogaster	Flybase617	1,023,087	29.45	80.85	83.85	85.86
Arabidopsis thaliana	Ensembl34	457,825	7.58	54.56	90.43	85.35
Zea mays	Ensembl34	1,099,124	7.12	65.29	86.24	91.08

Table S2: summary of annotated functional features and their distribution across transcript and protein isoforms (i.e. the level of resolution of the annotation) for the different species for which transcriptome annotations are included in tappAS.

Annotation level	Source	Category	No. feature occurrences	No. isoforms annotated
Transcript<br>11970 isoforms<br>7167 genes	ScanForMotifs	PAS	8511	5750 (48%)
	ScanForMotifs	3'UTR motifs	11797	5325 (44%)
	UTRscan/UTRsite	5'UTR motifs	325	315 (3%)
	UTRscan/UTRsite	uORF	7444	3045 (25%)
	RepeatMasker	Repeat regions	19269	7245 (61%)
	MiRWalk/miRbase + in-house scripts	3'UTR miRNA binding sites	106392	9474 (79%)
	clipDB + in-house scripts	RNA-binding sites (RBPs)	47821	7279 (61%)
Protein<br>10813 coding isoforms<br>7167 genes	In-house scripts	Nonsense-Mediated Decay (NMD)	329	329 (3%)
	PFAM-HMMER3	Domains	20973	9608 (89%)
	COILS + UniprotKB	Coiled coil	6669	2856 (26%)
	TMHMM + UniprotKB	Transmembrane regions	12543	2061 (19%)
	SignalP	Signal peptides	824	824 (8%)
	MOBIDB	Disordered regions	11256	5626 (52%)
	cNLS mapper + UniprotKB	Nuclear Localization Signals (NLS)	7599	4297 (40%)
	PSP + UniprotKB	Post-Translational Modifications (PTM)	100804	8506 (79%)
	UniprotKB	Compositional bias	2260	1480 (14%)
	UniprotKB	Motif	6579	2897 (27%)
	UniprotKB	Intramembrane	159	62 (0.6%)
	UniprotKB	Active site	1770	1168 (11%)
	UniprotKB	Binding	12790	3339 (31%)

Table S3: summary of annotation results for the mouse transcriptome of NPC and OPC primary cells. Number of features at the transcript and protein levels annotated are indicated, together with their database of origin and the percentage of isoforms in the transcriptome that contain them.

3’ UTR motif	p-value	Adj.p-value	No. varying genes (%)	p-value random set	Adj.p-value random set
GU-rich Destabilization Element	0.0024	0.05	197 (69.4%)	0.26	0.69
GU-Rich Element (GRE)	0.0281	0.33	243 (66.2%)	0.16	0.60

Table S4: ID-level FDA results for UTR motifs using the positional approach. Motifs with a p-value less than 0.01 are listed. Significance was assessed via Fisher’s Exact Test with Bonferroni-Hochberg multiple-testing correction. Results for a randomized set of RefSeq transcripts (N = 11,970) are reported as control.

miRNA	p-value	Adj.p-value	No. varying genes (%)	p-value random set	Adj.p-value random set
mmu-miR-335-3p	9e-4	0.46	61 (62.2%)	0.21	1
mmu-miR-590-3p	0.0059	0.77	83 (56.8%)	0.11	1
mmu-miR-880-3p	0.0071	0.77	21 (70%)	0.34	1
mmu-miR-7b-3p	0.0101	0.77	43 (60.5%)	0.38	1
mmu-miR-223-3p	0.0145	0.77	35 (61.4%)	0.74	1

Table S5: ID-level FDA results for miRNA binding motifs using the positional approach, top-5 ranked by adjusted p-value. Significance assessed via Fisher’s Exact Test with Bonferroni-Hochberg multiple-testing correction. Results for a randomized set of RefSeq transcripts (N = 11,970) are reported as control.

Feature	No. of DFI features	NPC favored	OPC favored	No. of tested features
3’ UTR motif	177	74	103	1640
5’ UTR motif	27	7	20	131
Active site	14	5	9	106
Binding motif	113	50	63	805
Coiled coil	82	43	39	866
Compositional bias region	41	13	28	279
Disordered region	210	119	91	1598
PFAM domain	345	135	210	759
Intramembrane protein	7	0	7	10
miRNA binding	1601	716	885	9627
Motif	75	32	43	596
PTM	947	401	546	7600
RBP binding	79	36	43	694
Signal peptide	24	2	22	117
Transmembrane domain	141	62	79	803
uORF	591	228	363	3118

Table S6: Whole-transcriptome summary of DFI results. For each functional annotation category, this table summarizes the number of features detected as significantly differentially included (DFI p-value < 0.05), the number of features whose inclusion is favored in each of the conditions (NPCs or OPCs) and the total number of tested features (regardless of whether they were positive or negative for DFI).
