# Supplementary material for: tappAS: a comprehensive computational framework for the analysis of the functional impact of differential splicing
Source: Genome Biol. 2020 May 18;21:119. doi: 10.1186/s13059-020-02028-w (PMC7236505; doi:10.1186/s13059-020-02028-w)
Supplement: Supplementary file 3 — Additional file 3. Review history. [file 13059_2020_2028_MOESM3_ESM.docx]

Review History

**First round of review**

**Reviewer 1**

**Were you able to assess all statistics in the manuscript, including the appropriateness of statistical tests used?**

Yes, and I have assessed the statistics in my report.

**Were you able to directly test the software?**

No

**Comments to author:**

This manuscript presents tappAS, a software for the analysis of isoform switches from gene expression (RNA-seq) data. Several tools are already available for identifying switches, but this one is original in that it combines statistics for the identification of switches to a set of functions intended to measure their functional impact.

tappAS has many interesting and novel features and is a potentially useful addition to the transcript analysis toolbox. However, the manuscript suffers from a lack of essential technical information and some weakly supported biological claims.

Major comments:

In the Functional Diversity Analysis (FDA) section, the authors present a first application of their software to a mouse dataset but fail to present their input data. Readers need to know how many libraries were used, library types, study design, any preprocessing/normalization step performed and how transcripts were quantified, using which reference transcriptome. This information is essential for understanding downstream analysis and should be provided at least briefly in Results. Currently, readers have to look for it in Online Methods (there is no Methods section in the main text) and, still, key data is missing. For instance I could not find sample numbers anywhere. From Fig 3C I gather that there are only 2 replicates in the NPC vs OPC experiment. This would be very low and out of bounds for differential analysis, which questions the validity of the remaining of the study. Why was this dataset chosen while there are so many well designed RNA-seq experiments available?

Moreover, I understand this part of the analysis is only qualitative. Therefore I do not agree with sentence "NMD-targeted isoforms were co-expressed with functional isoforms in our neural system, possibly regulating their abundance". The only thing that can be said from this presence/absence analysis is that both NMD-regulated and functional isoforms are expressed in the dataset. On the same line, P. 14 the authors speculate about functional consequences of variations in GRE motifs and miRNA binding sites. I find it premature as it is not backed by statistics. At this stage, the most likely explanation is that GREs and miRNA binding sites are just abundant motifs that occur in variable regions in any transcript dataset. The same applies to discussion P.15 on Fig 2C and 2D. Overall, I find the whole discussion on FDA P.13-15 and its conclusion "FDA successfully recapitulates the transcriptome's potential for AltTP-mediated functional diversity" to be misleading. Results are interpreted in terms of biological properties of the mouse neural system, whereas it remains to be shown whether a random sample of the same number of transcripts would not have shown the same type of events.

Analysis of the functional impact of differential polyadenylation on miRNA targets and PFAM domains lacks proper controls. Among 5 enriched miRNA target sites in Table S5, 4 come from unreliable mouse miRNAs with large numbers of predicted targets according to TargetScan: miR-590-3p, miR-335-3p, miR-880-3p, miR-7a-2-3p/7b-3p. Site enrichment for these miRNAs may just result from their large number of targets. In this light, the discussion P.28 about regulation of Rufy3 (a miR-590-3p target) seems quite speculative. Only one miRNA, mmu-miR-223-3p has few predicted targets, but it is the least significant in Table S5. Likewise, C2H2-type Zn finger motifs apparently associated to APA (P.27) are from one of the top 20 PFAM families. I am not surprised it turns out enriched in any gene list, if not properly weighted.

Since user-friendliness is advertised, one could expect more practical implementation details in the main text. For now, details are scarce and provided solely in suppl. methods. Information on dependencies & input file format would enable rapid evaluation of whether the software answers readers needs. Also: what are the recommendations for building the input transcript expression table, and should it be in TPM?

Minor comments:

IsoformSwitchAnalyzeR (https://doi.org/10.1093/bioinformatics/btz247) is a related software that should at least be discussed and compared qualitatively to tappAS.  

Table 1:
- in metrics with equations: explain all variables n, g etc..
- In "Total Isoform usage Change": is it the same n in all 3 sums?

P.3 "and Alternative Transcription Start Sites (ATSS), jointly referred to as Alternative Transcript Processing (AltTP). "
> I do not think alternative start sites belong to alternative processing.

P. 9 the raw numbers of labels/annotation per species does not say much. Could you use #labels/transcript instead? If I understand correctly there should be >10 labels/transcript in mouse (2.5M labels / 200k transcripts). Am I right?

P. 15. "variation rate", "rate of variation":
The use of "rate" here is incorrect to convey the feature's presence or absence in some isoforms.

P. 17. DTL analysis
Same comment as for chapter on FDA: insert necessary information on input data and study design at beginning of chapter.

P. 17: "revealing that a fraction of the posttranscriptional regulation was independent of changes in total gene expression"
> Isn't that implicit in "posttranscriptional regulation" ?

P. 66.
"Co-DFI is computed for each pair of features detected as DFI in at least 5 genes"
> Is a P-value computed? How?

Fig 3C: Is each dot a measure from 1 sample ?  

P. 19: "using DE genes as background."
Please explain how this background is incorporated in the statistics and P-value.

P. 19. "NLS and PTMs were among the most significantly enriched motifs"
Introduce acronym NLS here.

P. 73. Experimental setup in murine neural cells.
This part is not detailed enough.
 
Suppl Fig 4: Some terms are shown with -log10(adj P)<1, this means adj P<0.1
This is not significant and should be removed

P. 21 "In summary, the Differential module in tappAS can be successfully used to study..."
I am not sure authors can claim "success" in this mostly descriptive chapter, even though some of the functions altered by differential usage are consistent with the literature.

P. 25. "tappAS' unprecedented ability to provide functional category-level differential inclusion evaluations.."
I find the word "unprecedented" a bit overinflated here. This chapter just shows the program performs as intended in reporting differential events.

P. 25: "frequent associations between NLS and phosphoserine residues"
> Fig S7A/B shows these features are adjacent. Thus how significant is the association?  

P. 31. "Ctnnd1 (Figure 3C) shows the presence of "
I guess it is Fig S3.

P. 31 "cytoplasmic retention p120 "
> of p12O

P.32 "across cell types and were statistically"
delete "and"

Fig 6a: it is unclear which isoforms lack a NLS.

Fig 6d: use "Ctnnd1/P120" in Western blot label

**Reviewer 2**

**Were you able to assess all statistics in the manuscript, including the appropriateness of statistical tests used?**

Yes, and I have assessed the statistics in my report.

**Were you able to directly test the software?**

Yes

**Comments to author:**

The author of this article systematically proposed that alternative splicing, coding ability (CDS), UTR length changes and APA site changes should be systematically annotated at the transcript (isoforms) level. The research used long read long sequencing of RNA to obtain the full length isoforms. However, there are still some issues that need to be greatly improved and addressed.
1、     There are 7 analyses and 7 metrics used in tappAS, how do the authors to evaluated the contributions of these analyses and metrics, and which one is the dominate contribution and which is the minor one. Besides, why selected these seven novel metrics, are they independent for each other.
2、     In module1, the functional diversity were evaluated by presence varying and positional varying. Do the length of the varying region in these two types affect the function? Moreover, the position (UTR/CDS/end of gene body/middle of gene body) effect of these two types may be different in isoform, which should been addressed.
3、     In module2 and module3, the stages (T1, T2 and T3) and conditions (Cond1 and Cond2) are listed in Figure 1, what's the different between them, why use different identifiers in different situations
4、     There is too little in the discussion section. And why the authors selected 7 metrics as the criteria for isoform functional analysis should be emphatically discussed, and the theoretical evidence is important.
5、     Although this text has a certain role in promoting the functional analysis of A, it lacks a theoretical basis and explanation
6、     The author presented the experimental verification based on mouse data, and the DIU of Ctnnd1 and Mbnl1 is analyzed, which proved the validity of this framework to some extent, but should it be able to provide fully statistics analysis of isoforms in these data sets, so that readers can intuitively understand how many events can be detected in each analysis of the experimental data used in this study. It will also be an important reference for readers.
7、     Experimental design for obtaining more comprehensive structural information of full-length transcripts have been reported, for example, isoforms full-length PolyA sequencing. The author should consider increasing the development of this part in the manuscript.

**Authors Response**

**Point-by-point responses to the reviewers’ comments:**

Reviewer reports:

Reviewer #1: This manuscript presents tappAS, a software for the analysis of isoform switches from gene expression (RNA-seq) data. Several tools are already available for identifying switches, but this one is original in that it combines statistics for the identification of switches to a set of functions intended to measure their functional impact.

tappAS has many interesting and novel features and is a potentially useful addition to the transcript analysis toolbox. However, the manuscript suffers from a lack of essential technical information and some weakly supported biological claims.

Major comments:

In the Functional Diversity Analysis (FDA) section, the authors present a first application of their software to a mouse dataset but fail to present their input data. Readers need to know how many libraries were used, library types, study design, any preprocessing/normalization step performed and how transcripts were quantified, using which reference transcriptome. This information is essential for understanding downstream analysis and should be provided at least briefly in Results. Currently, readers have to look for it in Online Methods (there is no Methods section in the main text) and, still, key data is missing. For instance I could not find sample numbers anywhere. From Fig 3C I gather that there are only 2 replicates in the NPC vs OPC experiment. This would be very low and out of bounds for differential analysis, which questions the validity of the remaining of the study. Why was this dataset chosen while there are so many well designed RNA-seq experiments available?

*Thank you for the remark. We apologize for the lack of details in the data description. We have now added a description of the data at the beginning of the results section. However, please note that the same dataset is used to demonstrate all of tappAS functionalities throughout the paper.*

*Specifically, we used a Pacbio dataset from a mouse neural differentiation system previously published by our group. Importantly, although tappAS can be used with any type of transcriptomics data, the analytical framework is particularly meaningful when transcripts are identified with long-read sequencing technologies, as these provide full-length transcript sequences. Long read sequencing -in contrast to short-reads- warrants accurate detection of transcript contiguity, which is the foundation of an annotation strategy based on motif and domain identification. For this reason, we illustrate tappAS with a long-read based transcriptome database. As PacBio’s lower sequencing depth compromises obtaining a reliable estimation of expression values, it is currently best practice to use Illumina sequencing to provide sufficient quantification power and estimate expression of the long-read defined transcripts. We have previously shown that this method yields improved quantification estimates [1], as only transcripts that are actually expressed are being used as reference for mapping. Unfortunately, not many replicated PacBio datasets were available at the time when these analyses were performed, although this is likely to change in the future due to the rapid cost-reduction in long-read sequencing technologies. However, we believe that the limited replication of the dataset used in this study is not a major issue when it comes to illustrating software usage, which is the main goal of this work. The intention of our paper is not to provide a detailed study of mouse neural cell development biology or to accurately model this process, but to use this dataset to demonstrate tappAS functionalities and for the creation of functional hypothesis than can then be experimentally validated. To further prove tappAS usefulness, we have performed such validations for two candidate genes, Ctnnd1 and Mbnl1. Therefore, we believe the provided dataset to be sufficient to demonstrate software usage.*

*That being said, we have illustrated tappAS´ functionalities in a different dataset with a higher replication level for the purpose of this review. This is a dataset of CD4 and CD19 cells in Type 1 Diabetes patients that was previously analyzed by our group [2] and includes 80 individuals. Please note that this is a dataset where Illumina RNA-seq data was mapped to the reference transcriptome, hence using no long-read data. However, we did use a novel quantification approach also developed by our group [3], shown to do a better job in quantifying isoform expression. We are attaching a report containing the results of this analysis to this letter. As you can observe, tappAS returns significant results in all types of analysis modules and highlights functional variation in genes associated to immune cell types. Interestingly, we identified some AltTEM patterns also found in our murine neural dataset, such as a significant fraction of DIU genes not being differentially expressed, enrichment of DIU and DE genes in relevant biological processes (also detecting some with preferential transcriptional or post-transcriptional regulation), global differences in 3’UTR length between cell types, preference in the inclusion of functional features for one of the cell types (CD4-cells) and an overall small total usage change in differentially included features, with some exceptions. These results suggest global common characteristics in the functional impact of AltTEM, although, naturally, the specific functional elements enriched in each biological system were different.*

*Since this analysis is part of a larger, ongoing study, we prefer not to publish these results in the present manuscript. However, we hope that these additional results serve to earn the reviewer’s confidence in the power of our method to study the functional aspects associated to transcript variants.*

Moreover, I understand this part of the analysis is only qualitative. Therefore I do not agree with sentence "NMD-targeted isoforms were co-expressed with functional isoforms in our neural system, possibly regulating their abundance". The only thing that can be said from this presence/absence analysis is that both NMD-regulated and functional isoforms are expressed in the dataset.

*We thank the reviewer for this remark. By the indicated sentence we wanted to highlight that in the studied transcriptome we do not observe any gene expressing only NMD transcripts. However, we agree with the reviewer than the FDA data only indicates that genes that express NMD isoforms also express functional counterparts, but no regulatory inference can be drawn from the data. We have rephrased the sentence to (Page 13):*

*Among them, nonsense-mediated decay (NMD) was the transcript-level feature with the highest rate of varying (95%), indicating that, in our neural transcriptome, nearly all genes generating NMD candidate isoforms also possess functional counterparts.*

*However, we could ask if we detect any evidence of NMD being used as a mechanism to regulate the functionality of a gene. For instance, if the transcriptional output for a given gene is shifted from a fully functional major isoform in one condition to an NMD major isoform in the other condition. This cannot be answered with our FDA, but actually, tappAS can easily address this question through a DFI analysis where NMD is taken as a feature. We have now performed this analysis, given the reviewer’s point on NMD, and as an additional demonstration of tappAS’s versatility as framework for functional analysis of transcript variants.*

*Running tappAS’ DFI analysis, we obtained 38 genes for which there was a significant (Q-value < = 0.05) isoform switch towards NMD isoforms (see Figure R1). These genes included published examples of auto-regulation through inclusion of cryptic exons leading to NMD isoforms like Tardbp/TDP-43 [4]. We then focused on the genes in which this isoform switch was major, and obtained only one result, Slc25a36. As can be seen in panel B), the NMD isoform of Slc25a36 went from ranking second in the NPC condition to being the most expressed transcript in the OPC condition with the functional counterpart behaving in an opposite way.*

*The richness of the functional annotation layers provided by tappAS is illustrated in panels C and D. In the genomic view of the transcripts, panel C), we show that the NMD isoform includes a cryptic exon flanked by PAR-CLIP sites that indicate binding of Srsf3, a well-known splicing factor and proto-oncogene involved in the inclusion and skipping of exons in different transcripts [3]. The effect on the CDS of the inclusion of this cryptic exon is displayed in panel D; the inclusion leads to a Premature Termination Codon (PTC) and the NMD flagging, as the PTC is more than 50 nucleotides usptream of the most 3’ Exon-Junction of the isoform. Moreover, using the DE module of tappAS we observed that Srsf3 was significantly downregulated in the oligodendrocyte condition (panel E). Therefore, from this analysis, we can speculate on the role of Srsf3 in the oligodendrocyte differentiation and in controlling NMD forms of Slc25a36. Such hypothesis is amenable to experimental follow-up and has been reached thanks to tappAS unparalleled capacity to integrate DE, DIU ‘classical’ expression analysis approaches with comprehensive functional annotation layers through the DFI module.*

*Figure R1. NMD analysis using tappAS’ DFI analysis.*

*On the same line, P. 14 the authors speculate about functional consequences of variations in GRE motifs and miRNA binding sites. I find it premature as it is not backed by statistics. At this stage, the most likely explanation is that GREs and miRNA binding sites are just abundant motifs that occur in variable regions in any transcript dataset. The same applies to discussion P.15 on Fig 2C and 2D. Overall, I find the whole discussion on FDA P.13-15 and its conclusion "FDA successfully recapitulates the transcriptome's potential for AltTP-mediated functional diversity" to be misleading. Results are interpreted in terms of biological properties of the mouse neural system, whereas it remains to be shown whether a random sample of the same number of transcripts would not have shown the same type of events.*

*We found the reviewer's comments very interesting. We would like to highlight that tappAS was designed to address and facilitate the answer of these kind of issues, providing methods and functional annotation. More specifically, at the ID level, the Functional Diversity Analysis provides the user a prioritized list of elements based on their relative enrichment in differentially included regions (Fisher’s Exact test is used in this case to provide an estimation of significance) among the isoforms expressed in the evaluated transcriptome. FDA is not intended to assess if these enrichments hold in other tissues or species. Therefore, we are at no point claiming that the highlighted enrichments are exclusive to the neural transcriptome.*

*In order to address the issues reported by the reviewer more specifically, we ran the FDA analysis for miRNA annotation using a random set of isoforms from the RefSeq mouse transcriptome (Figure R2). We found no common enriched features with mouse neural transcriptome results (A). Moreover, we ran the same analysis for the total set of RefSeq human isoforms, neither finding overlap between enriched miRNA binding sites (B), supporting the neural signal found by FDA in example data.*

*Figure R2. tappAS screenshot for FDA analysis at miRNA level using as input transcriptome A) a random set of mouse transcript isoforms from the RefSeq reference transcriptome and B) the whole RefSeq human transcriptome.*

Analysis of the functional impact of differential polyadenylation on miRNA targets and PFAM domains lacks proper controls. Among 5 enriched miRNA target sites in Table S5, 4 come from unreliable mouse miRNAs with large numbers of predicted targets according to TargetScan: miR-590-3p, miR-335-3p, miR-880-3p, miR-7a-2-3p/7b-3p. Site enrichment for these miRNAs may just result from their large number of targets. In this light, the discussion P.28 about regulation of Rufy3 (a miR-590-3p target) seems quite speculative. Only one miRNA, mmu-miR-223-3p has few predicted targets, but it is the least significant in Table S5. Likewise, C2H2-type Zn finger motifs apparently associated to APA (P.27) are from one of the top 20 PFAM families. I am not surprised it turns out enriched in any gene list, if not properly weighted.

*The referee raises an important issue that is the abundant predictions typically associated to microRNA. We are aware of this and we carefully designed our microRNA annotation strategy accordingly. First, we controlled for miRNA evidence: we parsed the miRBase database in order to define our set of high confidence miRNAs by including only those entries with the following experimental evidence: cloned, Northern, PCR, RT-PCR, qRT-PCR, 5’RACE, RTPCR, in-situ, qPCR, miRAP cloned, 3’RACE, insitu, RACE, miRAP, primer-extension, RAKE, obtaining a white list with just 512 miRNAs. This annotation strategy is now better described in the methods section (page 56). In particular, for the indicated microRNA the evidence was:*

*mmu-miR-335-3p - experimental; cloned, Illumina mmu-miR-590-3p - experimental; cloned*

*mmu-miR-880-3p -> experimental; cloned, 454, Illumina
mmu-miR-7b-3p > experimental; Illumina*

*mmu-miR-223-3p > experimental; cloned, Illumina*

*Moreover, we performed a comprehensive miRNA-binding site filtering: Following previous evaluations [6, 7], we applied a filtering approach for miRNA binding sites based on the number of sources reporting the association. This intersection method allowed to decrease the false positive rate and thus, increase the specificity. For a mRNA-miRNA interaction to be reported, the association had to be predicted by a minimum of 5 methods which had to include Targetscan, miRanda, and mirWalk, the last one providing transcript coordinate information for miRNA binding sites. This procedure is described in the Methods section. For example, in mouse this approach reduced by 92% the number of binding sites for whole RefSeq transcriptome reported by 12 prediction methods, going from 33,298,719 to 2,480,531 isoform-miRNA associations*

*Finally, and to specifically address the reviewer’s concern, we checked whether the microRNAs reported as enriched by the FDA were particularly biased forwards microRNAs with a high number of targets in our dataset. The microRNAs identified by our analysis spanned a wide range*

*of targets number within the distribution of targets in our neural dataset and	had a relatively
low number of targets when the full reference transcriptome (both RefSeq and Ensembl) were considered (Figure R3). We concluded there was not a bias in the significant microRNAs.*

*Figure R3. Distribution of the number of targets for the 512 experimentally validated miRNAs in the Neural transcriptome defined by PacBio (upper panel), the mouse RefSeq reference transcriptome provided by tappAS (medium panel) and the mouse Ensembl reference transcriptome provided by tappAS (lower panel).*

*Regarding C2H2-type zinc finger domains, we believe the reviewer’s concerns are valid. This is why we have carefully checked the annotation frequency of this particular feature, as well as the number of domains annotated in APA-regulated and non APA-regulated genes. Doing so, we obtain the following table:*

|  | *Domain annotated  in APA-regulated  gene* | *Domain annotated  in non APA-  regualted gene* | *Total* |
| --- | --- | --- | --- |
| *C2H2 domain is DFI* | *54* | *0* | *54* |
| *C2H2 domain is NOT DFI* | *36* | *1* | *37* |
| *Total* | *90* | *1* | *91* |

*We have therefore observed that, contrary to what the reviewer pointed out, C2H2-type zinc finger PFAM domains are consistently annotated in APA-regulated genes in our transcriptome, with only one exception. We agree that this scenario does not provide sufficient information to link C2H2 differential inclusion to APA, given that there are practically no annotations of this domain type outside of APA-regulated genes. However, we believe that this high annotation frequency among APA-regulated genes in comparison to other PFAM domains (Supplementary Figure 8) is not trivial. We consider that this finding points to a key role of APA regulation in C2H2-domain containing zinc finger proteins, even if alternative polyadenylation cannot be associated to changes in the inclusion of the domain via AltTEM mechanisms. Therefore, we have changed that section of the original discussion in our paper to capture this idea better and added the annotation frequencies in APA genes to our results (Page 24):*

*For example, C2H2-type zinc fingers were by far the most frequently DFI domain within the subset of APA genes (Supplementary Figure 8B). This is a consequence of nearly all C2H2 domain annotations in our transcriptome being present in APA-regulated genes (90 of 91, 98.9%) and suggests that UTR regulation might be an important component of C2H2 zinc-finger proteins.*

Since user-friendliness is advertised, one could expect more practical implementation details in the main text. For now, details are scarce and provided solely in suppl. methods. Information on dependencies & input file format would enable rapid evaluation of whether the software answers readers needs. Also: what are the recommendations for building the input transcript expression table, and should it be in TPM?

*We thank the reviewer for this valuable addition to our manuscript. To better explain tappAS user-friendliness, we have added a new paragraph to the introduction highlighting what we believe to be our application’s main strong points in this regard: 1) low number of dependencies, 2) few input files and 3) easy-to-use graphical user interface (GUI) (Page 5). To complete this description, we have incorporated a new figure (now Figure 1) containing two panels, one explaining the process of project creation, the inputs required and how this process accommodates both short and long-read data, and another panel detailing the main features of tappAS GUI.*

*Regarding the reviewer’s questions on input format, tappAS has been built around count-based methods such as DEXSeq and is therefore designed to work on count data. However, we provide flexibility regarding normalization methods: users may choose to pre-normalize their data, or to do so at the time of project creation using the TMM method [8]. We have detailed this in the above-mentioned section of the manuscript (Page 11).*

Minor comments:

IsoformSwitchAnalyzeR is a related software that should at least be discussed and compared qualitatively to tappAS.

*We thank the reviewer for bringing this paper to our attention. We have acknowledged IsoformSwitchAnalyzeR as relevant, previously published software bringing functional perspective into isoform-level analyses. We have added this citation, together with other additional splicing tools, to the Introduction (Page 4) and Discussion (Page 31) sections of the paper, highlighting the most important characteristics that differentiate our tool from previous resources.*

*Page 4: Other attempts at integrating isoform-level analyses with a functional perspective, such as IsoformSwitchAnalyzeR [7], are limited to transcript expression switches, leave other aspects of isoform regulation unaddressed and fully rely on external tools to provide functional insight.*

*Page 31: While many methods to statistically evaluate isoform expression differences exist, they either lack evaluation of full-length isoforms [37–40] or are limited to specific alternative processing events, such as APA [107–109], intron retention [110, 111] or alternative TSS [112– 114]. Furthermore, methods aiming to provide functional interpretation to isoform usage are either limited to coding features [41, 43, 54] or do not provide built-in functional data [55].*

Table 1:

- in metrics with equations: explain all variables n, g etc..

- In "Total Isoform usage Change": is it the same n in all 3 sums?

*We thank the reviewer for pointing this out. Variables in equations for Feature Inclusion, Distal PolyA Site Usage (DPAU), total usage change and weighted UTR length are now explained in detail Table 1. Total Isoform Usage Change formula metrics have also been described in methods (i.e. we have explicitly stated the meaning of n and that it is the same in all three sums).*

P.3 "and Alternative Transcription Start Sites (ATSS), jointly referred to as Alternative Transcript Processing (AltTP). " I do not think alternative start sites belong to alternative processing.

*The reviewer makes the fair remark that an alternative start site cannot be considered exclusively as an alternative processing of an existing transcript.*

*tappAS evaluates the changes in expression and functional impact of transcript variants. These variants are the consequence of alternative splicing, alternative transcription start sites and alternative transcription termination sites. We found convenient to have a general term to describe join the processes that result in this diversity and chose AltTP, however we understand*

*that this is not an established term and might not be 100% accurate. We have changed the term to Alternative Transcript Expression Mechanisms (AltTEM), which we believe is a more suitable choice.*

P. 9 the raw numbers of labels/annotation per species does not say much. Could you use #labels/transcript instead? If I understand correctly there should be >10 labels/transcript in mouse (2.5M labels / 200k transcripts). Am I right?

*Thank you for highlighting the need of reporting the number of features annotated per isoform. We have included the mean number of features in both the text and supplementary table (Page 52).*

*Regarding results, the RefSeq87 mouse transcriptome contains 106060 transcripts, getting an average of 24 labels/transcript.*

P. 15. "variation rate", "rate of variation":

The use of "rate" here is incorrect to convey the feature's presence or absence in some

isoforms.

*We apologize for the confusion in the term variation rate. When talking about variation rate, we are not referring solely to the presence of absence of features in isoform, but actually to the number of genes for which the feature is varying in inclusion across transcripts, over the number of genes with annotation to that feature:*

*varying rate (feature f) = (# genes with transcripts with differential inclusion on f) /
(# genes with annotation to f)*

*To clearly donte to this concept we have rephrased this rate as “varying rate” or “rate of varying” in throughout the manuscript.*

P. 17. DTL analysis

Same comment as for chapter on FDA: insert necessary information on input data and study

design at beginning of chapter.

*As indicated above, we have now added more details on the dataset both at the beginning of the Results and in the relevant Methods section.*

P. 17: "revealing that a fraction of the posttranscriptional regulation was independent of changes in total gene expression” -isn't that implicit in "posttranscriptional regulation"?

*We thank the reviewer for this comment, and we would like to take the opportunity to clarify our message. Here we use “changes in total gene expression” as a proxy to transcriptional regulation mechanisms, that is the mechanisms that regulate the rate and the amount of transcripts produced for a given gene (for instance by enhancer mediated recruitment of co-activator factors or RNApol-II to a promoter). By post-transcriptional regulation, we mean the production of alternative isoforms through Alternative Splicing (AS), Alternative PolyAdenylation (APA), and Alternative Transcription Start Sites (ATSS), now jointly referred to in this manuscript as Alternative Transcript Expression Mechanisms (AltTEM).*

*Although the classical trend is to describe these two sets of mechanisms as independent and sequential we now know that they can be coupled and interdependent [10]. This is also true for alternative splicing because commitment to a particular splicing step occurs co-transcriptionally even though the actual intron excision is completed after release of pre-mRNA from the gene [8]. Coupling is achieved by coordinating the rates of elongation and processing of the transcript [8] which allows for cross-regulation between both sets of mechanisms. The rate of transcript elongation influences splicing outcomes according to the “window of opportunity” kinetic model. Conversely, post-transcriptional factors can induce transcriptional pause [10, 11].*

*So, accepting DE genes as a good proxy of genes under active transcriptional regulation and DIU genes as a good proxy of genes for which there is a clear indication of alternative post-transcriptional regulation our software allows to understand to which extent genes are*

*simultaneously -or not- regulated- by both mechanisms. We found	intriguing to understand if
a differentially expressed gene is also frequently differentially spliced, what would suggest that both processes concur to control the gene’s biological function. Or, on the contrary, the two regulatory mechanisms operate indistinctively. Our data supports the second model for an important fraction of the DIU genes being not differentially expressed.*

*We have rephrased the sentence to capture this idea better (Page 16):*

*Interestingly, ~50% of DIU genes were not DE (Figure 4A), indicating that a relevant fraction of the post-transcriptional regulation was independent of changes in total gene expression.*

P. 66: "Co-DFI is computed for each pair of features detected as DFI in at least 5 genes" Is a P-value computed? How?

*TappAS’ co-DFI analysis currently lacks an evaluation of significance, i.e. it does not return a p-value for the association of differential inclusion of feature pairs, as the null-distribution for such test has a complicated definition. However, we consider this analysis to be sufficient to qualitatively evaluate co-occurrence, firstly given that Differential Feature Inclusion is statistically tested beforehand and that multiple-testing corrections are performed for this analysis. In addition, we incorporate the 5-gene threshold to ensure that qualitative associations reported in our co-DFI table are less likely to be spurious. Finally, we note that co-DFI analysis was developed in order to quantitatively rank pairs of features that present co-occurrence and prioritize them for further validation.*

Fig 3C: Is each dot a measure from 1 sample?

*We have modified the legend in figure 3C (now 4C) for clarity (Page 21):*

*C) From left to right, gene, transcript and protein-level expression charts for the Cntn1 gene in the two conditions in our system (NPC, OPC, each containing two replicates -squares indicate replicate expression, points indicate mean replicate expression), and tappAS graphical representation of its transcript-level annotation.*

P. 19: "using DE genes as background."

Please explain how this background is incorporated in the statistics and P-value.

*When performing Functional Enrichment using a Fisher’s Exact Test in this section, we compared the frequencies of GO term annotations in the list of genes with significant Differential Isoform Usage (DIU) to the frequencies of GO term annotations in the list of DE genes. Hence, in this analysis setup, we expect significance (i.e. adjusted p-value < 0.05) to be assigned to functions that are significantly more abundant in DIU genes (AltTEM regulated) than in DE genes (transcriptionally-regulated). Therefore, this analysis aims to detect the functions mainly regulated via AltTEM.*

*We have now rephrased this to “using DE genes as comparing list” (Page 17).*

P. 19. "NLS and PTMs were among the most significantly enriched motifs" Introduce acronym NLS here.

*Thank you for pointing this out. NLS acronyms have now been introduced as suggested.*

P. 73. Experimental setup in murine neural cells.
This part is not detailed enough.

*As stated in our manuscript, this study used already-published data from Tardaguila et al. [1], were experimental setup as well as processing details have been extensively described in other. For the shake of manuscript length, we have chosen not to elaborate on this, although following the reviewer’s comments, we have carefully checked that the number of cell types and replicates included in the study are sufficiently clear throughout the paper. In addition, we have added more details to Methods’ “Experimental setup in murine cells” section.*

Suppl Fig 4: Some terms are shown with -log10(adj P)<1, this means adj P<0.1 This is not significant and should be removed

*We disagree with the reviewer in this. We believe that “significant” is a relative concept that relates to an arbitrary, i.e. user-defined, significance threshold. We agree that stating that a result is significant and not specifying the significance value would be problematic. However, please note that we do not claim significance, but instead provide the actual significance estimation values (adjusted p-value) for the reader’s evaluation.*

P. 21 "In summary, the Differential module in tappAS can be successfully used to study..."

I am not sure authors can claim "success" in this mostly descriptive chapter, even though some

of the functions altered by differential usage are consistent with the literature.

*We have adapted language to better reflect the scope of the claims made in this section (page 19):*

*In summary, these examples demonstrated how the Differential module in tappAS can be used to study the balance between differential gene expression and AltTEM in a transcriptome analysis, and to illustrate their combined biological effects.*

P. 25. "tappAS' unprecedented ability to provide functional category-level differential inclusion evaluations.."

I find the word "unprecedented" a bit overinflated here. This chapter just shows the program performs as intended in reporting differential events.

*We have removed the word “unprecedented” to better reflect the scope of the claims made in this section (Page 22):*

*These results bring to light tappAS’ ability to provide functional category-level differential inclusion evaluations, as well as to capture the condition-dependent impact of AltTEM on individual functional elements.*

P. 25: "frequent associations between NLS and phosphoserine residues"

Fig S7A/B shows these features are adjacent. Thus how significant is the association?

*As we said in previous reviewer point, tappAS’ co-DFI analysis currently lacks an evaluation of significance, i.e. we do not produce a p-value for the association of differential inclusion of feature pairs, therefore the association between NLS and phosphoserine residues is reported as frequent and not as significant. Specifically, NLS-Phosphoserine residues were ranked as the most frequently associated feature pair in our neural transcriptome with a total of 66 associations (58 co-inclusions and 8 mutual exclusions between studied conditions). This type of association is in agreement with previous studies reporting the relevant role of PTMs in the masking of NLSs [12– 14].*

P. 31. "Ctnnd1 (Figure 3C) shows the presence of "
I guess it is Fig S3.

*We have checked and fixed the error. This sentence now points to both Figure 3C and Supplementary Figure 3, since they are both relevant in this context.*

P. 31 "cytoplasmic retention p120 "

of p12O

*We have corrected the error.*

P.32 "across cell types and were statistically"
delete "and"

*We have corrected the error.*

Fig 6a: it is unclear which isoforms lack a NLS.

*We have edited Figure 6A (now 7A) and added additional information to the legend in order to clearly state which isoforms lack the NLS.*

Fig 6d: use "Ctnnd1/P120" in Western blot label

*We have modified Western blot labels in Figure 6D (now 7D).*

Reviewer #2: The author of this article systematically proposed that alternative splicing, coding ability (CDS), UTR length changes and APA site changes should be systematically annotated at the transcript (isoforms) level. The research used long read long sequencing of RNA to obtain the full length isoforms. However, there are still some issues that need to be greatly improved and addressed.

1、	There are 7 analyses and 7 metrics used in tappAS, how do the authors to evaluated the contributions of these analyses and metrics, and which one is the dominate contribution and which is the minor one. Besides, why selected these seven novel metrics, are they independent for each other.

*Thank you for this remark, as it is very important to us that our manuscript clearly reflects the methodologies implemented in tappAS. There are indeed several analysis and metrics implemented in tappAS. Each one addressed one aspect of the analysis of the functional analysis of alternative isoforms. We cannot say one metric dominates over others, as they are complementary. We agree with a later comment by this reviewer that this has not been sufficiently explained and discussed in the manuscript and we have now added a new long paragraph at the discussion talking about these metrics and analysis and how they are combined in the tappAS framework (Pages 29-30).*

2、	In module1, the functional diversity were evaluated by presence varying and positional varying. Do the length of the varying region in these two types affect the function? Moreover, the position (UTR/CDS/end of gene body/middle of gene body) effect of these two types may be different in isoform, which should been addressed.

*The length of the feature is considered when we use the “positional varying” mode, since it compares the exact genomic position of elements having the same ID. At present we do not establish a model to relate varying region length and functional impact, although visual inspection in each case will reveal the magnitude of the difference. In the case of presence varying, by definition, the varying statement implies that the feature is totally excluded in one isoform, therefore the length of the varying region is the length of the feature, and therefore its function should be absent.*

*Regarding the second question, we would like to clarify two relevant aspects of isoform annotation. First, we don’t map features simply through a blind overlap of genomics coordinates, but we annotate individually each isoform UTR and CDS with their corresponding functional categories. In relation to that, we would like to point out that all feature categories, except repetitive elements, can be only annotated in one specific region: CDS (NLS, PFAM...) or UTR (miRNAs, UTRmotifs...), but not to both regions. Considering these two aspects, one feature cannot change from being located at the CDS to the UTR, even their genomic coordinates match. Following the example, if the CDS is altered and the region coding the feature is now part of the UTR the feature won’t be annotated anymore and will be called as varying, both using the “positional” and the “presence” mode. Oppositely, this novel untranscribed region can be potential to accommodate new regulatory elements such as ARE or miRNA binding sites, which will be labelled also as varying (feature inclusion).*

3.In module2 and module3, the stages (T1, T2 and T3) and conditions (Cond1 and Cond2) are listed in Figure 1, what's the different between them, why use different identifiers in different situations

*Since tappAS accepts both two-condition and time-course experimental designs, the labels reflect these possibilities. Therefore, T1, T2 and T3 correspond to time-points, and Cond1 and Cond2, to conditions.*

4.There is too little in the discussion section. And why the authors selected 7 metrics as the criteria for isoform functional analysis should be emphatically discussed, and the theoretical evidence is important.

*Please refer to our answer to Q1. We have now added two new paragraphs at the Discussion of the paper (page 29 and 30) where we discuss the rationale of our approach and the utility of the different tappAS metrics.*

5.Although this text has a certain role in promoting the functional analysis of A, it lacks a theoretical basis and explanation

*Thank you for this remark, as it was important for us to establish the importance of the functional analysis at the isoform level. The reviewer’s comment indicates that this was not sufficiently motivated. We have now included the following paragraph in the discussion to explain the basis of the implemented approaches (Page 29):*

*The overall concept behind FIT is that transcript variants of the same gene imply sequence differences that may encode functional elements. By modifying these sequences, either by the inclusion or exclusion of exons, or by lengthening or shortening of UTRs, transcripts incorporate or exclude functional elements that are responsible for functional differences among isoforms. Therefore, the study of the functional impact of alternative isoform expression requires the study of the differential inclusion of these functional elements. This contrasts with the traditional approach to functional assessment of differential splicing that basically restricts to the GO/PATHWAY - based functional enrichment analysis of spliced genes, and requires both of an isoform-resolved annotation of functional sites and of novel methods to systematically handle positional features and evaluate functional isoform differences.*

6、	The author presented the experimental verification based on mouse data, and the DIU of Ctnnd1 and Mbnl1 is analyzed, which proved the validity of this framework to some extent, but should it be able to provide fully statistics analysis of isoforms in these data sets, so that readers can intuitively understand how many events can be detected in each analysis of the experimental data used in this study. It will also be an important reference for readers.

*We thank the reviewer for bringing this to our attention. To improve manuscript readability and better reflect the extent of the functional results output by our software, we have incorporated a supplementary table (Supplementary Table 6) summarizing Differential Feature Inclusion (DFI) analysis results for each feature category. Regarding DIU and DE results, we note that Supplementary File 1 (.tsv), which contains the DIU and DE status for transcripts, genes and CDS for each gene contained in our analysis, was provided with our initial submission.*

7、	Experimental design for obtaining more comprehensive structural information of full-length transcripts have been reported, for example, isoforms full-length PolyA sequencing. The author should consider increasing the development of this part in the manuscript.

*Thank you for this remark. This is an interesting aspect, but as far as we know there are not functional motifs described for polyAs and typically, polyAs are not part of the sequenced transcript. However, note that tappAS relies on an isoform-resolved functional annotation file as input for functional information. Theoretically, if information of polyA length is available, users could edit the gff3 annotation file to include this information and label this as polyA. In this case the FDA would be able, for example, to capture differences in length among transcripts, and the being annotated as part of the 3’UTR, the length of the polyA would contribute to the UTR shortening/lengthening analysis. In the discussion of the paper, we highlight that tappAS has the flexibility to allow for user’s provided annotation and we give as an example protein-protein interaction sites. We have now also mentioned the PolyA length (page 31) in the discussion as possible user-defined annotation element.*

*References:*

*Tardaguila M, de la Fuente L, Marti C, Pereira C, Pardo-Palacios FJ, del Risco H, et al. SQANTI: extensive characterization of long-read transcript sequences for quality control in full-length transcriptome identification and quantification. Genome Res. 2018. doi:10.1101/gr.222976.117.*

*Newman JRB, Conesa A, Mika M, et al. Disease-specific biases in alternative splicing and tissue-specific dysregulation revealed by multitissue profiling of lymphocyte gene expression in type 1 diabetes. Genome Res. 2017;27(11):1807–1815. doi:10.1101/gr.217984.116*

*. Newman JRB, Concannon P, Tardaguila M, Conesa A, McIntyre LM. Event Analysis: Using Transcript Events To Improve Estimates of Abundance in RNA-seq Data. G3 (Bethesda). 2018;8(9):2923–2940. Published 2018 Aug 30. doi:10.1534/g3.118.200373*

*Sugai A, Kato T, Koyama A, Koike Y, Konno T, Ishihara T, et al. Non-genetically modified models exhibit TARDBP mRNA increase due to perturbed TDP-43 autoregulation. Neurobiol Dis. 2019;130:104534. doi:10.1016/j.nbd.2019.104534.*

*Ajiro M, Jia R, Yang Y, Zhu J, Zheng Z-M. A genome landscape of SRSF3-regulated splicing events and gene expression in human osteosarcoma U2OS cells. Nucleic Acids Res. 2016;44:1854–70. doi:10.1093/nar/gkv1500.*

*Sethupathy P, Corda B, Hatzigeorgiou A. TarBase: A comprehensive database of experimentally supported animal microRNA targets. RNA. 2005;12:192–7. doi:10.1261/rna.2239606.*

*Kuhn DE, Martin MM, Feldman DS, Terry A V., Nuovo GJ, Elton TS. Experimental validation of miRNA targets. Methods. 2008;44:47–54. doi:10.1016/j.ymeth.2007.09.005.*

*Robinson MD, Oshlack A. A scaling normalization method for differential expression analysis of RNA-seq data. Genome Biol. 2010;11:R25. doi:10.1186/gb-2010-11-3-r25.*

*Vitting-Seerup K, Sandelin A. IsoformSwitchAnalyzeR: analysis of changes in genome-wide patterns of alternative splicing and its functional consequences. Bioinformatics. 2019;35:4469–71. doi:10.1093/bioinformatics/btz247.*

*Bentley DL. Coupling mRNA processing with transcription in time and space. Nat Rev Genet. 2014;15:163–75. doi:10.1038/nrg3662.*

*Mayer A, Landry HM, Churchman LS. Pause & go: from the discovery of RNA polymerase pausing to its functional implications. Curr Opin Cell Biol. 2017;46:72–80. doi:10.1016/j.ceb.2017.03.002.*

*Niklas KJ, Bondos SE, Dunker AK, Newman SA. Rethinking gene regulatory networks in light of alternative splicing, intrinsically disordered protein domains, and post-translational modifications. Front Cell Dev Biol. 2015;3. doi:10.3389/fcell.2015.00008.*

*Wright PE, Dyson HJ. Intrinsically disordered proteins in cellular signalling and regulation. Nat Rev Mol Cell Biol. 2015.*

*Bah A, Forman-Kay JD. Modulation of intrinsically disordered protein function by post-translational modifications. J Biol Chem. 2016;291:6696–705.*

**Second round of review**

**Reviewer 1**

I thank the authors for their careful answers and complementary analysis addressing my concerns. I accept the explanation as to why such a limited dataset was used as test case.

A few remaining comments:

P.6 l.7 average:2 4 features
-> 24 features

P.6 l.24: use defined
-> user defined

P.6 L 31: ... which positionally maps tappAS functional annotations onto newly-defined transcriptomes
-> I don't understand this. "TappAS functional annotation" is supposed to be the result of the whole process. Then how can it be used at this stage?

P. 13 "nearly all genes generating NMD candidate isoforms also possess functional counterparts."
-> I may suggest instead: "nearly all genes generating NMD candidate isoforms also express functional counterparts."

I accept the explanation given to my comment on the possible non-significance of GRE/miRNA site enrichment (Fig R2). However it seems this was not transferred into the manuscript. P-values and results of randomized controls should be provided to readers.

I accept authors' explanation about the significance of miRNA target enrichment with APA. Thank you for Figure R3.

The C2H2 zinc fingers explanation looks convincing too, except that the ratio of APA-regulated to non-APA-regulated genes should be given, so that the 90/1 ratio is meaningful.

P. 23 L30: polyadelination -> polyadenylation

The authors now appropriately cite IsoformSwitchAnalyzeRI, but I find the phrasing "Other attempts at integrating isoform-level ..." quite dismissive. This is not an attempt, this is a tool that works (and I am not its author!)

Thanks for the explanation about DE vs post-transcriptional regulation. Point taken.

**Authors Response**

**Point-by-point responses to the reviewers’ comments:**

Reviewer #1:

I thank the authors for their careful answers and complementary analysis addressing my concerns. I accept the explanation as to why such a limited dataset was used as test case.

A few remaining comments:

- P.6 l.7 average:2 4 features -> 24 features

- P.6 l.24: use defined -> user defined

*Thank you for pointing these out. We fixed both typing errors in the new version of the manuscript.*

- P.6 L 31: ... which positionally maps tappAS functional annotations onto newly-defined transcriptomes -> I don't understand this. "TappAS functional annotation" is supposed to be the result of the whole process. Then how can it be used at this stage?

*The tappAS software performs functional analyses of Alternative Transcript Expression Mechanisms (AlTEM) using a set of functional features that are retrieved from databases and mapped to transcript isoforms using genomic positions. These are contained in a gff3 annotation file. Throughout the manuscript, we refer to this file as tappAS «functional annotation». Using the IsoAnnotLite tool, functional annotation files created by the tappAS developer’s team and available in tappAS website can be used to annotate any user-defined transcriptome. To do this, IsoAnnotLite uses gff3 genomic positions to find positional matches in the unannotated transcriptome, and transfers the functional features to these novel transcript isoforms. This is the process that we refer to when talking about «positionally map tappAS functional annotations onto newly-defined transcriptomes». To make this clearer we have now slightly modified the sentence:

"For this purpose, tappAS toolkit includes the IsoAnnotLite algorithm, which positionally maps tappAS functional annotation labels onto newly-defined transcriptomes."*


- P. 13 "nearly all genes generating NMD candidate isoforms also possess functional counterparts." -> I may suggest instead: "nearly all genes generating NMD candidate isoforms also express functional counterparts."
 *We thank the reviewer for this suggestion. We have changed the sentence accordingly in the new version of the manuscript.*

- I accept the explanation given to my comment on the possible non-significance of GRE/miRNA site enrichment (Fig R2). However it seems this was not transferred into the manuscript. P-values and results of randomized controls should be provided to readers.

*Thank you for pointing this out. To better illustrate the reliability of our analyses, we have now completed tables S4 and S5 (Additiona_file_1.pdf ) including the p-values of ID-level FDA analyses performed as a control with a random set of RefSeq transcripts, for GREs and miRNAs.*


- I accept authors' explanation about the significance of miRNA target enrichment with APA. Thank you for Figure R3.

- The C2H2 zinc fingers explanation looks convincing too, except that the ratio of APA-regulated to non-APA-regulated genes should be given, so that the 90/1 ratio is meaningful.

*As suggested, we have included the APA/non-APA ratio in this part of the manuscript for clarity, but would like to remind that the absolute numbers of APA and non-APA regulated genes are stated at the beginnig of these results section.*

- P. 23 L30: polyadelination -> polyadenylation

*We fixed this typing error.*

- The authors now appropriately cite IsoformSwitchAnalyzeRI, but I find the phrasing "Other attempts at integrating isoform-level ..." quite dismissive. This is not an attempt, this is a tool that works (and I am not its author!)

*We thank the reviewer for this remark. In order to avoid coming across as dismissive, which is far from our intention, we have rephrased this sentence:

"Other software tools aimed at integrating isoform-level analyses with a functional perspective, such as IsoformSwitchAnalyzeR [55], are limited to transcript expression switches, leave other aspects of isoform regulation unaddressed and fully rely on external tools to provide functional insight."*

- Thanks for the explanation about DE vs post-transcriptional regulation. Point taken.
